# Cortical thickness and white matter microstructure predict freezing of gait development in Parkinson’s disease

**DOI:** 10.1038/s41531-024-00629-x

**Published:** 2024-01-09

**Authors:** Fabin Lin, Xinyang Zou, Jiaqi Su, Lijun Wan, Shenglong Wu, Haoling Xu, Yuqi Zeng, Yongjie Li, Xiaochun Chen, Guofa Cai, Qinyong Ye, Guoen Cai

**Affiliations:** 1 Department of Neurology, Center for Cognitive Neurology, Institute of Clinical Neurology, Fujian Medical University Union Hospital, Fuzhou, 350001 China; 2 Fujian Institute of Geriatrics, Fujian Medical University Union Hospital, Fuzhou, 350001 China; 3 Fujian Key Laboratory of Molecular Neurology, Fujian Medical University, Fuzhou, 350001 China; 4 Department of Neurosurgery, Fujian Medical University Union Hospital, Fuzhou, 350001 China; 5 Shengli Clinical Medical College, Fujian Medical University, Fuzhou, 350001 Fujian China; 6 School of Basic Medical Sciences, Fujian Medical University, Fuzhou, 350001 China; 7 College of Information Engineering, Guangdong University of Technology, Guangzhou, 510006 Guangdong China

**Keywords:** Parkinson's disease, Predictive markers

## Abstract

The clinical applications of the association of cortical thickness and white matter fiber with freezing of gait (FoG) are limited in patients with Parkinson’s disease (PD). In this retrospective study, using white matter fiber from diffusion-weighted imaging and cortical thickness from structural-weighted imaging of magnetic resonance imaging, we investigated whether a machine learning-based model can help assess the risk of FoG at the individual level in patients with PD. Data from the Parkinson’s Disease Progression Marker Initiative database were used as the discovery cohort, whereas those from the Fujian Medical University Union Hospital Parkinson’s Disease database were used as the external validation cohort. Clinical variables, white matter fiber, and cortical thickness were selected by random forest regression. The selected features were used to train the support vector machine(SVM) learning models. The median area under the receiver operating characteristic curve (AUC) was calculated. Model performance was validated using the external validation cohort. In the discovery cohort, 25 patients with PD were defined as FoG converters (15 men, mean age 62.1 years), whereas 60 were defined as FoG nonconverters (38 men, mean age 58.5 years). In the external validation cohort, 18 patients with PD were defined as FoG converters (8 men, mean age 66.9 years), whereas 37 were defined as FoG nonconverters (21 men, mean age 65.1 years). In the discovery cohort, the model trained with clinical variables, cortical thickness, and white matter fiber exhibited better performance (AUC, 0.67–0.88). More importantly, SVM-radial kernel models trained using random over-sampling examples, incorporating white matter fiber, cortical thickness, and clinical variables exhibited better performance (AUC, 0.88). This model trained using the above mentioned features was successfully validated in an external validation cohort (AUC, 0.91). Furthermore, the following minimal feature sets that were used: fractional anisotropy value and mean diffusivity value for right thalamic radiation, age at baseline, and cortical thickness for left precentral gyrus and right dorsal posterior cingulate gyrus. Therefore, machine learning-based models using white matter fiber and cortical thickness can help predict the risk of FoG conversion at the individual level in patients with PD, with improved performance when combined with clinical variables.

## Introduction

Parkinson’s disease (PD) is a neurodegenerative disorder characterized by involuntary and uncontrollable movements, including tremors, rigidity, and difficulties with balance and coordination, frequently leading to freezing of gait (FoG)^1^. FoG is characterized by a transient or marked decrease in forward foot movement despite the intention to walk (4th International Workshop on Freezing of Gait). There is a correlation between FoG prevalence and disease duration, with a higher prevalence in individuals with a disease duration of more than 9 years (64.6%) than in those with a disease duration of up to 5 years (37.9%)^2^. Severe FoG significantly decreases the health-related quality of life of patients with PD^3,4^. Therefore, developing a reliable and objective method to predict its occurrence is vital.

We can obtain valuable insights by understanding the pathophysiological basis of FoG conversion in PD. At present, the specific pathophysiological mechanisms of FoG are still unclear; however, studies have suggested that abnormal cortical and white matter fiber tracts play a role in its pathogenesis^5^. These abnormalities include disruptions to the connection between the frontal and parietal lobes^6^, changes in the microstructural integrity of the bilateral superior frontal gyrus^7^, and decreased white matter fiber tracts connecting the pedunculopontine nucleus^8^. We speculate that cortical and white matter damage patterns can function as promising imaging evidence for predicting FoG. However, the results from longitudinal studies are scarce, and whether cortical and white matter damage patterns can predict the risk of PD progression remains unknown.

Machine learning is a vital branch of artificial intelligence that uses data-driven methods to facilitate the automatic extraction of latent patterns and regularities from vast datasets by computer systems via algorithm and model training. This, in turn, facilitates decision-making and predictions among researchers^9^. Machine learning has recently emerged as a powerful tool in the field of medical imaging, including computer vision techniques that can autonomously recognize and analyze image features. This technology can help extract advanced features and patterns from complex neural imaging data^10^. Furthermore, owing to its remarkable multidimensional analytical capabilities, machine learning can be used for classification at the individual level in the field of medical imaging^11,12^. However, neuroimaging-based techniques are rarely used to detect and predict FoG in PD. Therefore, in the present study, we developed a machine learning-based model that used white matter fiber data from diffusion tensor imaging (DTI), cortical thickness data from T1 magnetic resonance imaging (MRI), and clinical variables to predict the risk of FoG at the individual level in patients with PD.

## Results

### Baseline characteristics

**Fig. 1:**
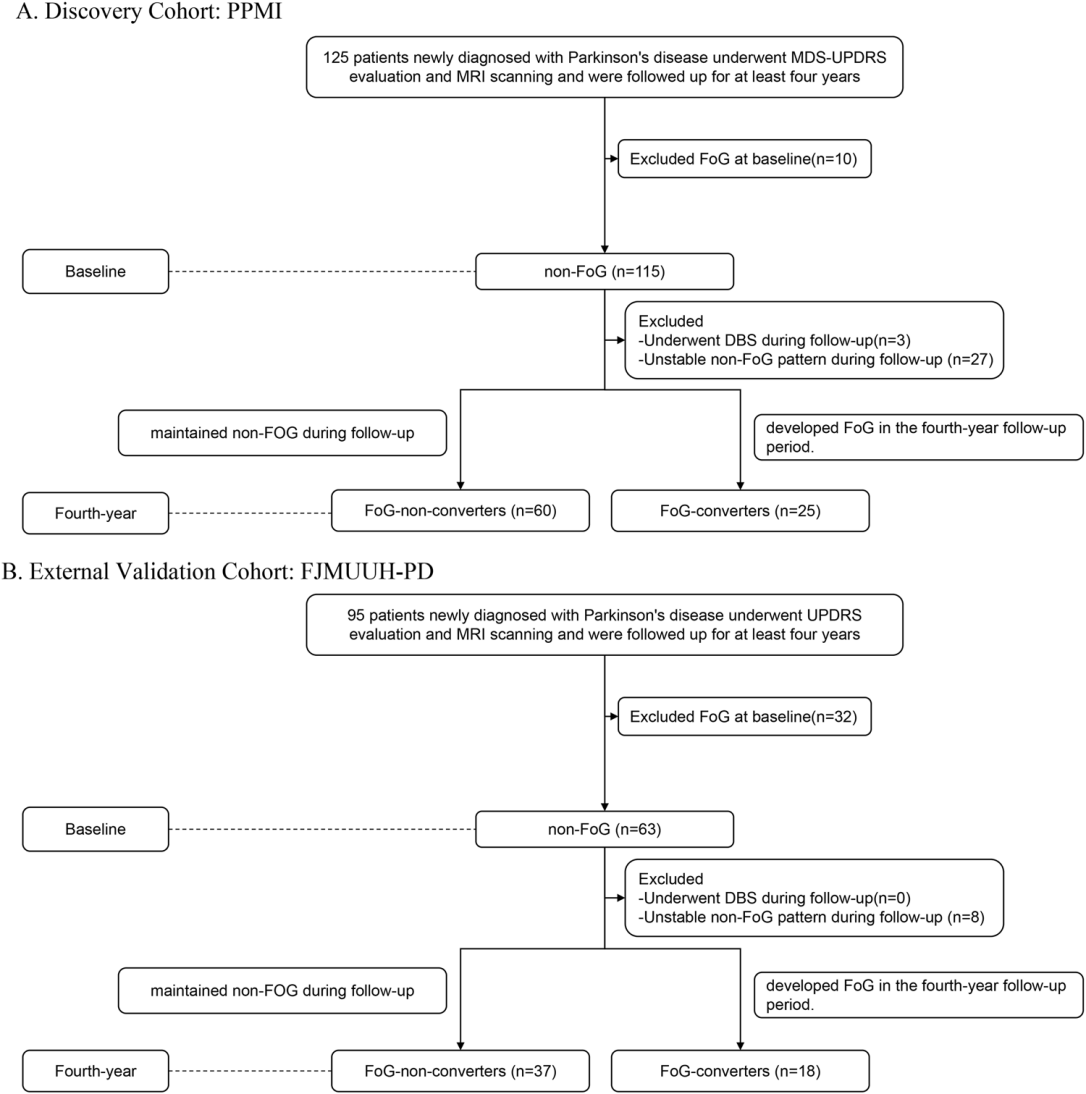
Flowchart depicting the patient inclusion process. **A** PPMI database and **B** FJMUUH-PD database. PPMI Parkinson’s Progression Marker Initiative, FJMUUH-PD Fujian Medical University Union Hospital Parkinson’s Disease, PD Parkinson’s disease, UPDRS Unified Parkinson’s Disease Rating Scale, MDS-UPDRS Movement Disorders Society-Unified Parkinson’s Disease Rating Scale, DBS deep brain stimulation, MRI magnetic resonance imaging, FOG freezing of gait.

Two cohorts were used in this study: discovery and external validation cohorts. The discovery cohort was obtained from the Parkinson’s Progression Marker Initiative (PPMI) database, whereas the external validation cohort was obtained from the Fujian Medical University Union Hospital Parkinson’s Disease (FJMUUH-PD) database. Table 1 summarizes the demographic and clinical characteristics of the patients in the discovery and external validation cohorts. Supplementary Table 1 summarizes the demographic and clinical characteristics according to FoG evolution. Of the 125 patients newly diagnosed with PD underwent Movement Disorder Society Unified Parkinson’s Disease Rating Scale (MDS-UPDRS) evaluation and MRI scanning and were followed up for at least 4 years in the discovery cohort, 40 patients were excluded: 10 who developed FoG patten at baseline, three who underwent deep brain stimulation (DBS) during follow-up, and 27 with unstable non-FoG pattern during follow-up. (Fig. 1A). Finally, the discovery cohort comprised 85 patients with PD, 25 FoG converters (10 women and 15 men, age 62.1 ± 13.2 years; Supplementary Table 1), and 60 FoG nonconverters (22 women and 38 men, age 58.5 ± 9.3 years; Table 1). In the validation cohort, among the 95 patients newly diagnosed with PD underwent UPDRS evaluation and MRI scanning and were followed up for at least four years, 32 with FoG patten at baseline were excluded, 8 with unstable non-FoG pattern during follow-up were excluded (Fig. 1B). Finally, 55 patients, 18 FoG converters (10 women and 8 men, age 66.9 ± 8.7 years), and 37 FoG nonconverters (16 women and 21 men, age 65.1 ± 6.3 years) were included for analysis.

**Table 1:** Patient demographics and clinical characteristics.

Characteristic	Level	PPMI	FJMUUH-PD	*P*-value
		*n* = 85	*n* = 55	
Age at baseline^a^	–	59.6 (10.6)	65.7 (7.1)	**<0.001**
Sex (%)^b^	Female	32.0 (37.6)	26.0 (47.3)	0.259
	Male	53.0 (62.4)	29.0 (52.7)	–
Years of education^c^	–	16.0 [14.0,18.0]	9.0 [6.0,12.0]	**<0.001**
H&Y (%)^b^	<2	45.0 (52.9)	16.0 (29.1)	**0.005**
	≥2	40.0 (47.1)	39.0 (70.9)	–
MDS-UPDRS-I scores^c^	–	1.0 [0.0, 2.0]	\	\
MDS-UPDRS-II scores^c^	–	5.0 [2.0,7.0]	\	\
MDS-UPDRS-III scores^c^	–	18.0 [13.5,24.0]	\	\
UPDRS-I scores^c^	–	\	2.0 [1.0,4.0]	\
UPDRS-II scores^a^	–	\	9.6 (4.9)	\
UPDRS-III scores^c^	–	\	27.0 [18.0,36.0]	\
RBDSQ^c^	–	3.0 [2.0, 5.0]	2.0 [0.0,4.0]	**0.004**
MoCA^c^	–	28.0 [26.0,29.0]	21.31 (5.4)	**<0.001**
MoCA (%)^b^	≥26	67.0 (78.8)	14.0 (25.5)	**<0.001**
	<26	18.0 (21.2)	41.0 (74.6)	–

Bold values represent correlations that are statistically significant.

*PPMI* Parkinson’s Progression Marker Initiative, *FJMUUH-PD* Fujian Medical University Union Hospital Parkinson’s Disease, *PD* Parkinson disease, *H&Y* Hoehn–Yahr, *MDS-UPDRS* Movement Disorder Society Unified Parkinson’s Disease Rating Scale, *RBDSQ* REM sleep behavior disorder Screening Questionnaire, *MoCA* Montreal Cognitive Assessment.

^a^Normally distributed continuous variables were compared using independent *t*-tests, and the results were reported as means along with standard deviations (means ±standard deviations).

^b^Categorical variables were compared using Chi-square tests, and the results were reported as counts and percentages (*n* (%)).

^c^Non-normally distributed continuous variables were assessed using the Mann–Whitney *U* test, and the results were reported as medians, along with interquartile ranges (medians [interquartile ranges]).

### Feature selection

**Fig. 2:**
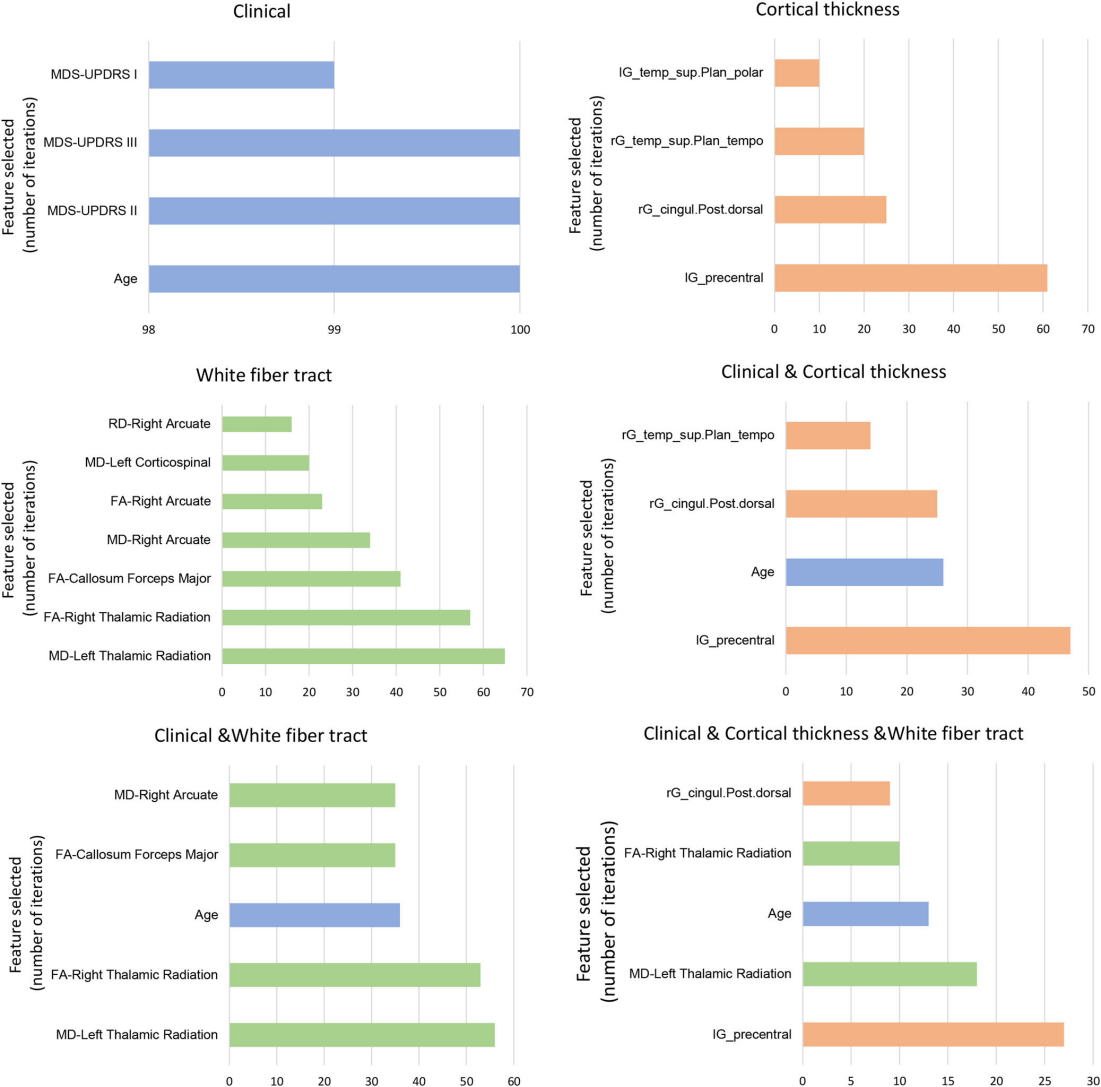
Bar graphs depicting the clinical variables, white matter fiber values, and cortical thickness features selected for the classification of freezing of gait converters and nonconverters according to the six feature combinations. MDS-UPDRS Movement Disorders Society-Unified Parkinson’s Disease Rating Scale, FA fractional anisotropy, RD radial diffusion, MD mean diffusivity, l left, r right, G_temp_sup-Plan_polar G_temp_sup-Planum_polare, G_temp_sup-Plan_tempo G_temp_sup-Planum_tempolare, G_cingul-post-dorsal G_posterior-dorsal.

**Fig. 3:**
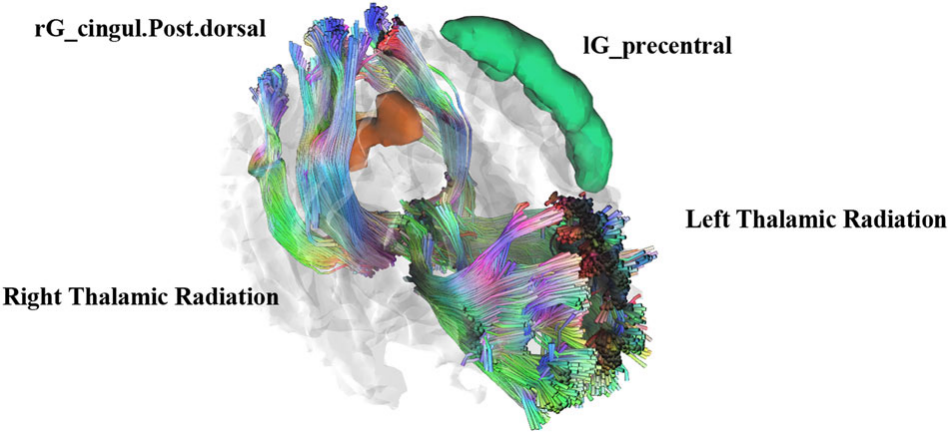
Visual images of selected cortical thickness and white matter fiber features for classification of freezing of gait converters and nonconverters.

The discovery dataset was divided in a 3:1 ratio into the training and test sets. Then, the training set was subjected to 10-fold cross-validation using the random forest model for feature selection. Among the clinical features, age and MDS-UPDRS-I, MDS-UPDRS-II, and MDS-UPDRS-III scores were selected. Among the features associated with white matter fiber, right thalamic radiation, callosum forceps major, right arcuate, left thalamic radiation, and left corticospinal tract were selected. Among the features associated with cortical thickness, rG_cingul-post-dorsal, lG_precentral, lG_temp_sup-Plan_polar, and rG_temp_sup-Plan_tempo were selected (Fig. 2). Table 2 and Fig. 3 presents the selected feature variables obtained from the training set of the discovery cohort. Supplementary Tables 2 and 3 summarizes the additional clinical, white matter fiber, and cortical thickness data for the FoG converters and nonconverters.

**Table 2:** Selected clinical, white matter fiber, and cortical thickness features according to the FoG evolution.

	PPMI				FJMUUH-PD			
Characteristic	FoG nonconverters	FoG converters	*P*-value	Adjusted *P*-value*	FoG nonconverters	FoG converters	*P*-value	Adjusted *P*-value*
	*n* = 60	*n* = 25			*n* = 37	*n* = 18		
Age at baseline^a^	58.5 (9.3)	62.1 (13.2)	0.163	/	65.1 (6.3)	66.9 (8.7)	0.382	/
UPDRS-I scores^b^	/	/	/	/	2.0 [1.0,4.0]	2.0 [0.0,4.3]	0.643	/
UPDRS-II scores^a^	/	/	/	/	9.6 (5.1)	9.61 (4.6)	0.994	/
UPDRS-III scores^b^	/	/	/	/	26.0 [18.0,34.0]	29.0 [17.75,38.3]	0.355	/
MDS-UPDRS-I scores^b^	1.0 [0.0, 1.0]	1.0 [1.0, 3.0]	**0.007**	/	/	/	/	/
MDS-UPDRS-II scores^b^	4.0 [2.0, 6.2]	6.0 [5.0, 9.0]	**0.003**	/	/	/	/	/
MDS-UPDRS-III scores^a^	18.2 (7.4)	22.4 (9.5)	**0.031**	/	/	/	/	/
**rG_cingul-post-dorsal**	**2.85** ± **0.17**	**2.73** ± **0.25**	**0.007**	**0.072**	0.39 ± 0.03	0.38 ± 0.03	0.004	0.110
**lG_precentral**	**2.51** ± **0.16**	**2.32** ± **0.26**	**<0.001**	**0.008**	1.13 ± 0.16	0.99 ± 0.2	0.005	0.103
**lG_temp_sup-Plan_polar**	**3.48** ± **0.24**	**3.27** ± **0.32**	**0.002**	**0.053**	0.51 ± 0.07	0.47 ± 0.1	0.018	0.181
rG_temp_sup-Plan_tempo	2.55 ± 0.13	2.49 ± 0.41	0.334	0.562	0.58 ± 0.1	0.5 ± 0.1	0.651	0.852
FA-right thalamic radiation	0.48 ± 0.03	0.46 ± 0.02	0.012	0.122	2.61 ± 0.43	2.79 ± 0.37	0.676	0.722
FA-callosum forceps major	0.61 ± 0.06	0.58 ± 0.16	0.276	0.480	2.29 ± 0.24	2.31 ± 0.17	0.493	0.540
FA-right arcuate	0.48 ± 0.04	0.46 ± 0.05	0.297	0.495	1.86 ± 0.17	1.86 ± 0.21	0.924	0.924
**MD-left thalamic radiation**	**0.72** ± **0.03**	**0.77** ± **0.05**	**<0.001**	**0.015**	**1.68** ± **0.18**	**1.74** ± **0.15**	**<0.001**	**0.004**
MD-left corticospinal	0.68 ± 0.03	0.71 ± 0.03	0.012	0.111	**2.13** ± **0.21**	**2.21** ± **0.17**	**0.004**	**0.010**
MD-right arcuate	0.7 ± 0.03	0.72 ± 0.06	0.060	0.252	**2.26** ± **0.22**	**2.31** ± **0.13**	**0.005**	**0.010**
RD-right arcuate	0.5 ± 0.04	0.53 ± 0.06	0.084	0.281	**1.84** ± **0.28**	**1.88** ± **0.32**	**0.016**	**0.024**

Categorical variables were compared using *χ*^2^ tests, and the results were reported as counts and percentages (*n* (%)).

Bold values represent correlations that are statistically significant.

*PPMI* Parkinson’s Progression Marker Initiative, *FJMUUH-PD* Fujian Medical University Union Hospital Parkinson’s Disease, *FoG* freeze of gait, *MDS-UPDRS* Movement Disorder Society Unified Parkinson’s Disease Rating Scale, *FA* fractional anisotropy, *MD* mean diffusivity, *RD* radial diffusion; *l* left, *r* right, *G_temp_sup-Plan_polar* G_temp_sup-Planum_polare, *G_temp_sup-Plan_tempo* G_temp_sup-Planum_tempolare, *G_cingul-Post-dorsal* G_posterior-dorsal.

^a^Normally distributed continuous variables were compared using independent *t* -tests, and the results were reported as means along with standard deviations (means ± standard deviations).

^b^Non-normally distributed continuous variables were assessed using the Mann-Whitney *U-*test, and the results were reported as medians, along with interquartile ranges (medians [interquartile ranges]).

*Adjusted by false discovery rate.

### Model performance and external validation

**Fig. 4:**
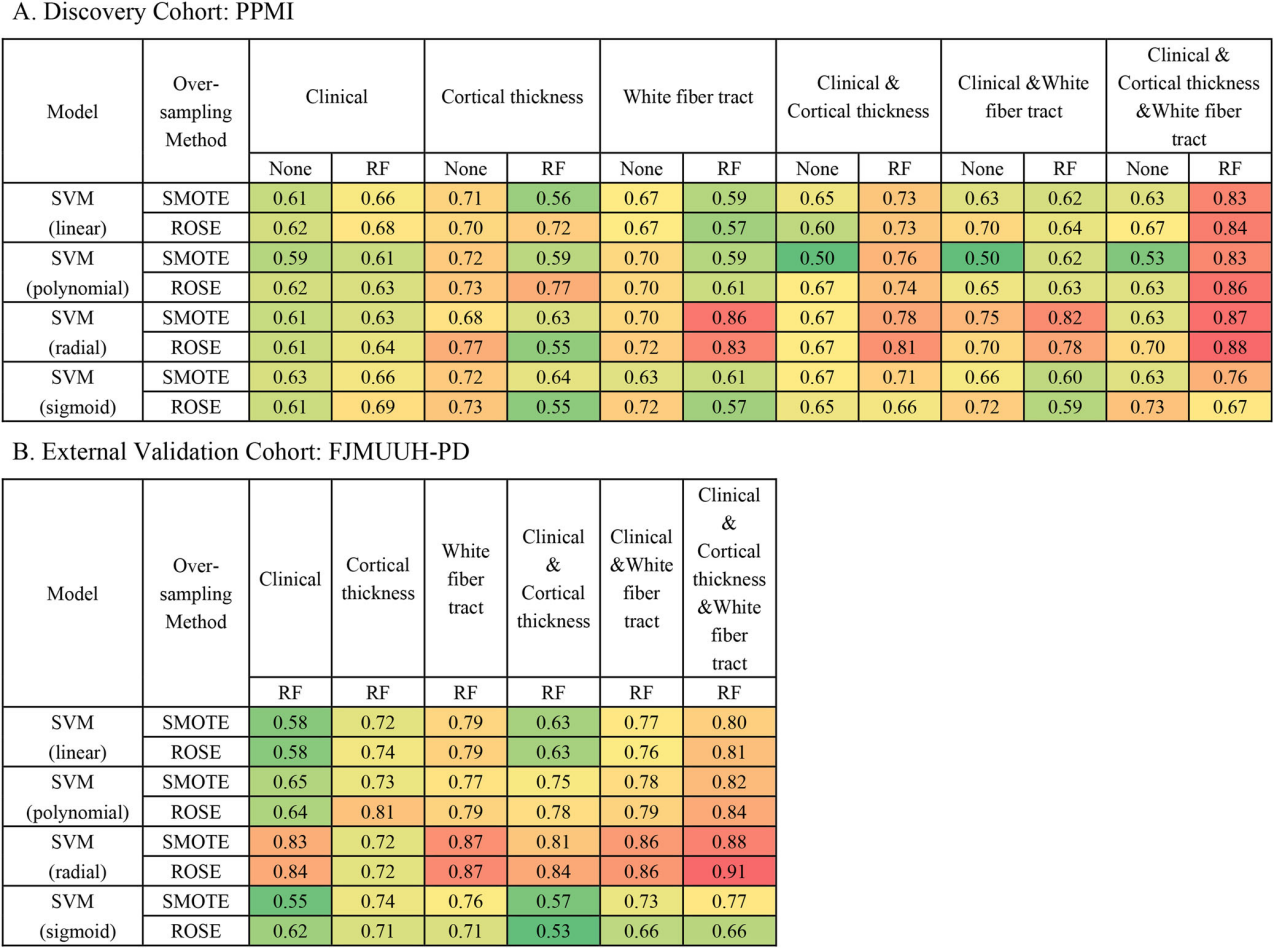
Heat maps of the area under the receiver operating characteristic curve from various machine-learning models for predicting the FOG conversion. **A** PPMI database and **B** FJMUUH-PD database. AUC values are presented as the median. ROSE random oversampling example, SMOTE synthetic minority oversampling technique, SVM support vector machine.

Figure 4 and Supplementary Tables 4 and 5 summarize the performance of the model in the discovery cohort and external validation set. Discovery cohort analysis revealed that the models trained using feature selection [the receiver operating characteristic curve (AUC), 0.567–0.922] consistently exhibited better performance than those without feature selection (AUC, 0.500–0.767). Therefore, we only used the models trained using feature selection to compare model performance in the discovery cohort and validate them in the external validation set.

**Fig. 5:**
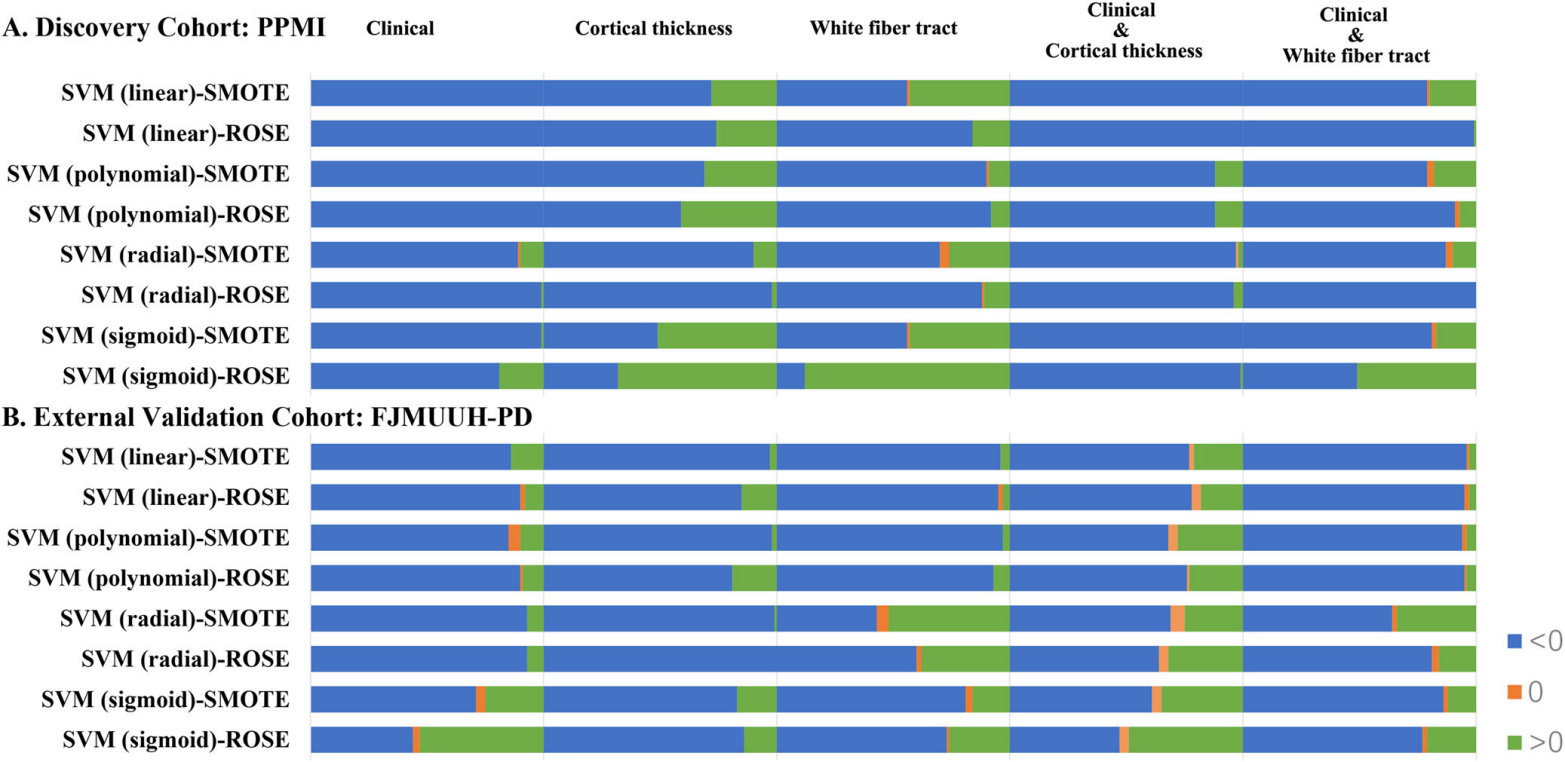
The horizontal bars depicting the results of pairwise comparisons of model performance for each iteration based on feature combinations. **A** PPMI database and **B** FJMUUH-PD database. When comparisons are made, a value greater than 0 indicates that the model for that variable (the clinical variable, the cortical thickness variable, the white matter fiber variable, the combined clinical and cortical thickness variable, and the combined clinical and white matter fiber variable) outperforms the model for the combination of clinical features, cortical thickness, and white matter fiber variables. A value equal to 0 indicates that the performance of the model for that variable matches the performance of the combined model for the clinical features, cortical thickness, and white matter fiber variables. Conversely, if the value is less than 0, it indicates that the performance of the combined model of clinical features, cortical thickness, and white matter fiber variables outperforms the model of this variable. ROSE random oversampling example, SMOTE synthetic minority oversampling technique, SVM support vector machine.

In the discovery cohort, the combined models trained using white matter fiber, cortical thickness, and clinical variables (AUC, 0.67–0.88) exhibited better performance than those trained using only clinical variables (AUC, 0.61–0.69), only cortical thickness variables (AUC, 0.55–0.77), only white matter fiber variables (AUC, 0.57–0.86), combined clinical and cortical thickness variables (AUC, 0.66–0.81) or combined clinical and white matter fiber variables (AUC, 0.59–0.82). Notably, support vector machine(SVM)-radial kernel models trained using random over-sampling examples(ROSE), incorporating white matter fiber, cortical thickness, and clinical variables exhibited better performance (AUC, 0.88). In the external validation cohort, the combined model trained using white matter fiber, cortical thickness, and clinical variables exhibited the best performance compared with the other models (AUC, 0.77–0.91), particularly the SVM radial kernel model using ROSE with AUC of 0.91. Combining the model results from the discovery and external validation cohorts, we observed that the SVM radial kernel model using ROSE exhibited the best and most consistent performance. Furthermore, the combined model trained using white matter fiber bundles, cortical thickness, and clinical variables exhibited consistently better performance than the other models, particularly the SVM model prepared using the ROSE oversampling method. In the discovery and external validation cohorts, pairwise comparisons revealed that the models trained using white matter fiber bundles, cortical thickness, and clinical variables consistently exhibited better performance more often than the combined model trained using white matter fiber bundles or cortical thickness and clinical variables (Fig. 5 and Supplementary Tables 6 and 7).

## Discussion

In clinical settings, group comparisons correlating cortical thickness and microstructural abnormalities in whole brain white matter with FoG in PD have limited applications. Therefore, in this study, we investigated whether the MRI data of cortical thickness and whole brain white matter microstructure and machine learning can help predict FoG development at the individual level in patients with PD. Our study suggested that using the ROSE oversampling method, SVM models, particularly the radial kernel model, trained using white matter fiber, cortical thickness data, and clinical variables, including the fractional anisotropy (FA) and mean diffusivity (MD) values of right thalamic radiation, age at baseline, cortical thickness of the IG_precentra and rG_cingul.Post.dorsal regions, can help predict FoG conversion at the individual level in patients with PD.

In previous studies, we observed that age, postural instability and gait disorder score, cognitive function, autonomic nervous function, sleep behavior, and fatigue are risk factors for PD-FoG^5,13^. However, apart from these clinical features, previous studies have also attempted to identify a responsible brain region or white matter tract that can be attributed to FoG conversion; nevertheless, most studies have focused on the differences in brain regions or white matter tracts between patients with PD who have already experienced FoG and those who have not experienced FoG. In the present study, we explored the potential brain regions and white matter tracts responsible for FoG conversion. We identified the right middle frontal gyrus and left postcentral gyrus as the responsible brain regions and the left and right thalamic radiations as the responsible white matter tracts. This highlights the essential role of age of occurrence.

In the feature importance analysis, we determined the pathophysiological features most relevant to FoG conversion, except for the precentral gyrus^14^, whose gray matter atrophy correlates with motor deficit severity^14^, the right dorsal posterior cingulate gyrus exhibited high importance and weight among the features associated with cortical thickness. The cingulate gyrus is part of the limbic system and is associated with human emotions and self-evaluation^15,16^. Arguably, previous studies have reported that the atrophy of the posterior cingulate gyrus is frequently associated with depression and anxiety^6,17^. An accepted explanation is that emotional load detracts from attentional resources in PD-FoG, in turn leading to sudden gait dysfunction^15,17,18^. Furthermore, the importance of the bilateral superior temporal gyrus (STG) has been emphasized when selecting clinical and white matter features, which may be associated with balance in previous studies^19^. Patients with PD with frequent falls have considerably lower gray matter volume and functional connectivity in the STG and inferior parietal lobule than those who were able to maintain balance^19^. The STG may be involved in processing vestibular information and postural functions; neuromodulator inhibition of the STG may result in impairments in self-motor perception, leading to dyskinesia^20,21^.

Among the features associated with white matter fiber, we observed that bilateral thalamic radiation exhibits high importance and weight to clarify FoG converters. Many studies have reported that thalamic radiation is associated with FoG in PD^18,22,23^. Marumoto et al. have reported that thalamic radiation is connected to the frontal motor areas of the thalamus, including supplementary motor areas, and that the FA characteristics of this white matter bundle can be used to predict gait speed^23^. Similarly, Lenfeldt et al. have reported that diffusion changes in the thalamus can help differentiate tremor dominance from postural instability and gait disorder phenotypes^24^. This finding is consistent with the ability of thalamic radiation to differentiate FoG converters from FoG nonconverters in the present study. Furthermore, callosum forceps major and right arcuate fasciculus play notable roles in white matter fiber tracts and white matter fiber tracts and clinical features. Many researchers consider the arcuate fasciculus to be a part of the superior longitudinal fasciculus (SLF), the frontotemporal segment. The SLF, similar to thalamic radiation, is significantly different in patients with PD with and without FoG^25^; this difference may be centered in the temporal bundle^6^, which coincides with the findings of our study. However, our study is the first to identify the role of the callosum forceps major in PD-FoG. The callosum forceps major connects the splenium of the corpus callosum to the occipital cortex. Previous studies have reported that the corpus callosum and its surrounding structures and occipital region are closely associated with PD-FoG^6,18,22,26^. Furthermore, the pressor region of the corpus callosum is closely associated with cognitive function in patients with PD^26^. Previous studies have considered gait an automatic motor task that does not require much cognitive involvement. However, recently growing evidence suggests the importance of cognition in gait, indirectly confirming the correlation between the callosum forceps major and PD-FoG^27,28^. However, in addition to these regions and white matter fiber tracts, other regions, such as, the corpus callosum, subfrontal gyrus triangle, subparietal marginal angular gyrus, cingulum hippocampus, cingulum cingulate, left corticospinal tract, callosum forceps minor, right uncinate, and SLF are associated with PD-FoG^6,23,25^. One plausible reason for this inconsistency is that in the present study, we differentiated patients who would develop FoG within 4 years of PD diagnosis from stable patients without FoG. This is essentially different from the cortical thickness and white matter tract abnormalities observed in patients with FoG compared with those without FoG in previous studies.

Nevertheless, the present study has some limitations, and the first one is its generalizability. Herein, we applied different machine-learning algorithms to 100 training and test sets; however, the results might vary if other machine-learning methods are applied to different cohorts. Therefore, the present results should be interpreted with caution. Second, we observed that the model trained using both cortical thickness and clinical variables and the model trained solely on cortical thickness, both exhibited seemingly unrealistic performance in the discovery phase. However, this outstanding performance was not observed for the external validation cohort. We think that this discrepancy may be attributed to overfitting, and therefore, caution should be exercised when interpreting the results of these two models. Furthermore, the MRI scanner and acquisition parameters employed in the external validation cohort differed from those employed in the discovery cohort. This difference might have led to non-biological bias in cortical thickness measurement^29^. SVM-radial kernel models trained using ROSE oversampling, incorporating white matter fiber, cortical thickness, and clinical variables, can successfully and individually predict FoG development in patients with PD. Our model has the potential for early identification of patients at risk of developing delayed FoG, thereby providing valuable guidance for clinicians in the prevention and intervention of FoG symptoms in PD patients. Future longitudinal multicenter studies with larger sample sizes and standardized measurements of cortical thickness and a whole-brain white matter microstructure should be performed to further validate the present findings.

## Methods

### Study design and participants

The data used in the study consisted of two cohorts, namely a discovery cohort and an external validation cohort.

The discovery cohort was obtained from the PPMI database^30^, an ongoing international multicenter longitudinal cohort. The inclusion criteria for this cohort were newly diagnosed patients with PD in the preceding year who did not undergo any treatment for it, with available clinical assessment and analyzable imaging data. The exclusion criteria were patients under four years of follow-up, with incomplete follow-up, those who underwent DBS surgery during the follow-up, and those with diagnosed neurodegenerative diseases other than idiopathic PD during the follow-up.

The external validation cohort was obtained from the FJMUUH-PD database. Patients with PD were diagnosed using the UK Brain Bank criteria^31^. The inclusion and exclusion criteria were the same as for the PPMI cohort. The participants underwent extensive clinical assessments and MRI scans at the beginning of the study and then followed up every 6 months for the 1st year, and every 20months for the next 4 years.

The PPMI cohort was registered on ClinicalTrials.gov (NCT01141023), and the Ethical Standards Committee for Human Subjects approved the participating sites before the start of the study. Written informed consent was obtained from all study participants. The Ethics Committee of Fujian Medical University Union Hospital approved the FJMUUH-PD cohort (No. 2019-014), and participants provided written informed consent before enrollment, adhering to the Declaration of Helsinki.

### FoG definition

UPDRS and MDS–UPDRS are widely used tools to assess motor functions and symptoms^32^. The tools have been validated in several populations with high sensitivity and specificity. Because FoG episodes are more common when medication is discontinued, clinical examinations and scans were performed in the morning when the participants were ‘off’ medication – at least 12 h after the last dopaminergic intake. For the external test cohort, we used the FJMUUH-PD database, with a larger number of individuals using the entire Unified Parkinson’s Disease Rating Scale (UPDRS) to assess PD symptoms, whereas the MDS-UPDRS has only been used in recent years, with fewer individuals with complete data. Therefore, on balance, the entire UPDRS scale was used for the test cohort. However, according to the recommendations of the MDS-UPDRS Revision Task Force^32^, it is generally accepted that the MDS-UPDRS shows high internal consistency with the entire UPDRS (Cronbach’s alpha = 0.79–0.93 across parts) and strong correlations with the original UPDRS (rho = 0.96). In addition, reliable factor structures were obtained for each part, supporting the use of sum scores for each part. Besides, we are not the only study to use both scales to assess PD symptoms. For instance, a study by Latourelle et al. also used two PD databases with these two different assessment scales for the prediction of PD motor progression^33^. This clinometric evidence supports the validity of the MDS-UPDRS to assess PD. Thus, it is reasonable to consider using the scores in your algorithm in a comparable manner. Therefore, the use of MDS-UPDRS scores in a similar manner in our algorithms is reasonable. While we acknowledge that MDS-UPDRS is considered a more precise assessment tool that may lead to improved machine model performance, in our study, UPDRS was not included in the combined model training (Fig. 2). As a result, its impact on the comparison of model performance between the discovery cohort and external test cohort is relatively minor. In the discovery cohort, patients with PD were defined as being (1) with FoG if their MDS–UPDRS 2.13 was >0 or 3.11 (off-med) was >0 or (2) without FoG (non-FoG) if their MDS–UPDRS 2.13 and 3.11 (off-med) were zero. In the external validation cohort, patients with PD were defined as being (1) with FoG if their UPDRS 2.14 was >0 or (2) without FoG (non-FoG) if their UPDRS 2.14 were zero. This study only included patients with PD who were initially not diagnosed with FoG. We conducted a four-year follow-up on these patients, assessing their FoG status at least once a year. PD Patients without FoG during the follow-up were classified as (1) “FoG nonconverters,” whereas if patients with PD developed FoG symptoms in the fourth year, they were classified as (2) “FoG converters”.

### MRI metrics in PPMI

The 1.5-Tesla MRI scanners from various manufacturers, including SIEMENS, Philips Medical Systems, and GE Medical Systems, were used to acquire T1Wis of 11 patients. The flip angle was 8–15°, and the matrix size was 192–516 pixels in the X and Y directions and 88–170 slices in the Z direction. The slice thickness was 1.0–1.5 mm, and the echo time (TE), inversion time (TI), and repetition time (TR) were 2.7–4.0 ms, 0 or 1000.0 ms, and 7.0–8.5 or 2400.0 ms, respectively. The 3.0-Tesla MRI scanners from the same manufacturers were used to acquire T1WIs of 74 patients. The flip angle was 9–13°, and the matrix size was 240.0–288.0 pixels in the X and Y directions and 152–192 slices in the Z direction. The slice thickness was 1.0–1.2 mm, and the TE, TI, and TR were 2.3–3.6 ms, 0, 450 or 900.0 ms, and 3.9–2300.0 ms, respectively. The 3.0-Tesla MRI scanner (SIEMENS TrioTim), with a flip angle of 90°, 64 gradient directions, and a matrix size of 1044.0 pixels in the X and Y directions and 65.0 slices in the Z direction, was used to acquire DTI images. The pixel size and slice thickness were 2.0 mm. The TE and TR were 88.0 and 670–9300.0 ms, respectively.

### MRI metrics in FJMUUH-PD

Brain imaging data were acquired using the GE 3.0-Tesla dual-gradient magnetic resonance scanner, and the sequences and scan parameters were as follows: a 3D cranial volume sequence (3D-BRAVO) obtained from high-resolution T1WI brain structure images, with TR = 8.7 ms, TE = 3.42 ms, TI = 400 ms, flip angle = 12°, matrix = 256 × 256, field of view = 240 × 240 mm, and 180 layers with a thickness of 1.1 mm. A spin echo-planar imaging sequence was used to obtain DTI scans for cross-sectional brain imaging, with the scanning level parallel to the anterior–posterior commissure. The DTI sequence required the following conditions: a TR of 6000 ms, TE of 65.7 ms, flip angle of 90°, matrix of 128 × 128, a field of view of 240 × 240 mm, 55 layers with a thickness of 3 mm, continuous scanning without spacing, b-values of 0 and 1000 s/mm^2^, and 16 nonlinear diffusion-sensitive gradient directions.

### Cortical thickness analysis

The surface-based morphometry analysis was performed using Statistical Parametric Mapping software (SPM12; http://www.fil.ion.ucl.ac.uk/spm/software/spm12), extended by the Computational Anatomy Toolbox (CAT12; http://dbm.neuro.uni-jena.de/cat/). The CAT12 default settings were used as described in detail in the manual (http://dbm.neuro.uni-jena.de/cat12/CAT12-Manual.pdf) to estimate the cortical thickness^34^. T1WI was registered with the Montreal Neurological Institute template, segmented into gray matter, white matter, and cerebrospinal fluid, and spatially normalized. A projection-based approach, which calculates the distance between the external cortical surfaces (the borders between the gray matter and cerebrospinal fluid), was used to estimate the left and right hemisphere cortical thicknesses. Finally, a 12-mm full width at the half maximum Gaussian kernel was used to smooth the cortical thickness.

### DTI analysis

Sequential automated steps were performed using MATLAB (2018b) with Automatic Fiber Bundle Quantification (AFQ), an open-source software developed by Yeatman’s team based on MATLAB that automatically identifies, quantifies, and analyzes white matter fiber pathways in the brain. The software automatically and efficiently extracts data on 20 major nerve fiber bundles across the brain and divides each into 100 isometric segments. Subsequently, FA and other diffusion tensor metrics are mapped to each segment to enable the accurate localization of abnormal changes in fiber bundle tensor imaging metrics^35^. AFQ is used to study white matter degeneration in mild cognitive impairment and its relationship with Alzheimer’s disease^36^. Before running AFQ, the path should be confirmed, and anterior commissure (AC)–posterior commissure (PC) lines should be aligned with midsagittal planes by entering the relevant codes in the command window. This procedure aligns the AC (located below the anterior front of the vault), PC (located below the posterior thalamus and above the midbrain), and midsagittal planes in the image. After alignment, the software extracts the FA, axial diffusion, radial diffusion, MD, and other structural characteristic data. The specific procedure and white matter fiber analysis parameters are described in the methodology section of the supplemental file.

### Feature selection and training of machine-learning models

A total of 96 combinations of models were trained, including four machine-learning methods and two oversampling methods, with and without feature selection. Potential features were identified from 17 clinical variables, 80 white fiber values, and 148 regional cortical thickness variables (Supplementary Table 8). The following combinations were used: (1) clinical variables, (2) white matter fiber values, (3) cortical thickness variables, (4) clinical variables and white matter fiber values, (5) cortical thickness and clinical variables, and (6) white matter fiber values and cortical thickness and clinical variables. We followed a feature selection approach using the random forest method to improve the predictive performance of the model. This method comprises several essential steps as follows. Initially, we randomly generated 100 training and divided the dataset into training and testing subsets, typically in a 3:1 ratio. Next, we used the 10-fold cross-validation process to ensure the robust evaluation of the model. The method was crucial because it could calculate feature importance. Within each cross-validation iteration, we trained a random forest classifier in the training subset to estimate the contribution of each feature to the predictive accuracy of the model. The computed feature importance accumulated across the cross-validation iterations. Features exceeding a predefined threshold, which was set at 50%, were deemed significant and subjected to further analysis. Next, the retained features were sorted based on their frequency of selection throughout the cross-validation process, ensuring that the most frequently selected and consistent features were prioritized.

### Model performance and external testing

We used the following four machine-learning methods: SVM with four different kernels (linear, polynomial, radial basis function (RBF), and sigmoid). SVMs are implemented using various kernels using the e1071 package in R(v 4.2.0)^37,38^. SVMs are powerful models for classification and regression that find the optimal hyperplane, which maximizes the margin between classes, thus effectively separating different class data points, and it has been used in our previous study^39^. SVMs use different kernels to shape their behavior. The linear kernel assumes linear separability and uses straight lines or hyperplanes to separate data; thus, it is suitable for linearly separable data. The polynomial kernel maps data to a higher-dimensional space using polynomial functions; thus, the method can capture complex decision boundaries. The RBF kernel is highly flexible and effectively models non-linear decision boundaries. Additionally, it is governed by a Gaussian-like kernel function. The sigmoid kernel introduces non-linearity through sigmoid functions and adds versatility to the behavior of the model. When dealing with imbalanced datasets in the present study, we considered two distinct oversampling methods: ROSE and synthetic minority over-sampling technique (SMOTE). ROSE improved class balance by replicating existing samples within the minority class, whereas SMOTE generated synthetic new samples by interpolating between samples in the minority class. These synthetic samples were generated based on feature relationships among neighboring samples that introduced greater diversity. The choice of method depended on dataset characteristics and specific requirements. ROSE was suitable for straightforward oversampling needs, whereas SMOTE was more appropriate when greater diversity and complex interpolation were desired. We opted for one of these methods or combined them to effectively address the issue of patient imbalance. We employed different data preprocessing strategies tailored to the specific requirements of each model. Specifically, we normalized the variables for the SVM model, ensuring they had a mean of zero and a standard deviation of one. This normalization improved the performance of SVM in handling the data effectively. Additionally, we considered the following two scenarios during modeling: one with feature selection and another without feature selection. Finally, we compared the performance of these two models with and without feature selection to determine the most suitable approach for our data and task. This flexible approach allowed us to gain a better understanding of the data and optimize the modeling outcomes. The use of the four machine-learning methods, cross-validation techniques, two oversampling approaches, and standardization methods have been reported in a previous study by Shin et al.^40^.

### Model performance and external testing

A dataset was initially divided into a training set and a test set, with a ratio of 3:1, during machine-learning performance analysis. The training set was used for model construction and parameter tuning, whereas the test set was reserved for the final evaluation of the performance of the model. Further, to select the best model and fine-tune hyperparameters, we followed the 10-fold cross-validation method to assess the model on the training set. Ensuring the independence of the test set from the training set and the cross-validation sets was crucial to guarantee the reliability of the final model evaluation. This workflow helped prevent overly optimistic estimations during evaluation because the test set remains separate from the model selection process. The machine-learning models were trained to predict the probability of FoG development for each patient in the test dataset, aiming to discover potential patient clusters.

Subsequently, we reconstructed and tested these probabilities in 100 resampled training and test datasets, evaluating the performance of each model using the area under the AUC. Owing to the skewed distribution of results in certain models, we used the median AUC as a performance metric. We compared the AUCs of models with different feature combinations and recorded the number of iterations in which model performance differed. Specifically, we conducted pairwise AUC comparisons in each iteration, tallying the frequency at which one model outperformed another, and calculated AUC differences between these pairs by performing paired t-tests. Hence, we could quantify performance disparities between the models and identify feature combinations that significantly affected model performance improvement. Within the 100 resampled datasets, we used the average feature importance and weights from random forest and SVM with a linear kernel to ascertain the predictive contribution of each feature to FoG occurrence. Finally, these models were applied to the external validation cohort to assess their generalizability and portability, with results showed based on the median AUC of the models.

### Reporting summary

Further information on research design is available in the Nature Research Reporting Summary linked to this article.

### Supplementary information

SUPPLEMENTAL MATERIAL

REPORTING SUMMARY

STROBE checklist

## Data Availability

The discovery cohort, Parkinson’s Progression Marker Initiative (PPMI) database, is available on Parkinson’s Progression Markers Initiative www.ppmi-info.org/data. The external validation cohort, Fujian Medical University Union Hospital Parkinson’s Disease (FJMUUH-PD) database, is available from the corresponding author upon request.

## Code Availability

R (v 4.2.0), CAT12, Automatic Fiber Bundle Quantification (AFQ), FSL (https://fsl.fmrib.ox.ac.uk), MATLAB R2018b (http://www.mathworks.com) and SPM12 in the study are open sources.
