# Supplementary material for: Cortical thickness and white matter microstructure predict freezing of gait development in Parkinson’s disease
Source: NPJ Parkinsons Dis. 2024 Jan 9;10:16. doi: 10.1038/s41531-024-00629-x (PMC10776850; doi:10.1038/s41531-024-00629-x)
Supplement: Supplementary file 1 — SUPPLEMENTAL MATERIAL [file 41531_2024_629_MOESM1_ESM.pdf]

Supplementary Material

Supplementary Table 1. Demographic and clinical characteristics according to FoG evolution.

Supplementary Table 2: Comparison of all cortical thickness variables according to FoG evolution.

Supplementary Table 3: Comparison of all white matter fiber variables according to FoG evolution.

Supplementary Table 4. Performances of machine learning models to predict in the test set of the PPMI cohort.

Supplementary Table 5: Performances of machine learning models to predict in the test set of the FJMUUH-PD cohort.

Supplementary Table 6. Pair-wise comparisons of model performances per iteration according to feature combination PPMI.

Supplementary Table 7. Pair-wise comparisons of model performances per iteration according to feature combination FJMUUH-PD

Supplementary Table 8: List of clinical, cortical thickness and white matter fiber variables used as features

Supplementary Table 1. Demographic and clinical characteristics according to FoG evolution.

Characteristic	Level	PPMI			FJMUUH-PD		
		FoG nonconverters n=60	FoG converters n=25	*P*-value	FoG nonconverters n=37	FoG converters n=18	*P*-value
Age(y)		58.5 (9.3)	62.1 (13.2)	0.163	65.14(6.3)	66.94(8.65)	0.382
Sex (%)	Female	22 (36.7)	10 (40.0)	0.965	16(43.2)	10(55.6)	0.391
	Male	38 (63.3)	15 (60.0)		21(56.8)	8(44.4)	
Years of education		16.0 [14.0, 18.0]	16.0 [14.0, 18.0]	0.977	6[6,11]	12[9,16]	0.002
H&Y (%)	<2	36 (60.0)	9 (36.0)	0.075	12(32.0)	4(22.0)	0.434
	≥2	24 (40.0)	16 (64.0)		25(68.0)	14(78.0)	
MDS-UPDRS-I scores		1.0 [0.0, 1.0]	1.0 [1.0, 3.0]	0.007	\	\	\
MDS-UPDRS-II scores		4.0 [2.0, 6.2]	6.0 [5.0, 9.0]	0.003	\	\	\
MDS-UPDRS-III scores		18.2 (7.4)	22.4 (9.5)	0.031	\	\	\
UPDRS-I scores		\	\	\	2[1, 4]	2[0, 4.25]	0.643
UPDRS-II scores		\	\	\	9.62(5.08)	9.61(4.63)	0.994
UPDRS-III scores		\	\	\	26[18, 34]	29[17.75, 38.25]	0.355
RBDSQ		3.0 [2.0, 5.0]	3.0 [2.0, 7.0]	0.583	2[0, 6]	2[0,3]	0.84
MoCA							
Visuoconstructional Skills		4.0 [3.0, 4.0]	4.0 [3.0, 4.0]	0.33	3[2, 4.5]	3[2,3]	0.394
Naming		3.0 [3.0, 3.0]	3.0 [3.0, 3.0]	0.357	3[3, 3]	3[3, 3]	0.485
Delayed Recall		4.0 [2.8, 5.0]	4.0 [2.0, 5.0]	0.792	1[0, 3]	2[1.75, 3.25]	0.097
Attention		6.0 [6.0, 6.0]	6.0 [6.0, 6.0]	0.848	5[5, 6]	5[4, 6]	0.916
Verbal Fluency		3.0 [2.8, 3.0]	3.0 [2.0, 3.0]	0.045	1[1, 2]	3.5[1.75, 6]	<0.001
Abstraction		2.0 [2.0, 2.0]	2.0 [2.0, 2.0]	0.371	1[0, 1]	1[0, 2]	0.319
Orientation		6.0 [6.0, 6.0]	6.0 [6.0, 6.0]	0.258	5[5, 6]	6[6, 6]	0.012
Total		28.0 [26.8, 29.0]	27.0 [25.0, 29.0]	0.295	20.27(5.29)	23.44(4.95)	0.038
MoCA (%)	≥26	50 (83.3)	17 (68.0)	0.199	6(16.2)	8(44.4)	0.024
	<26	10 (16.7)	8 (32.0)		31(83.8)	10(55.6)	

PPMI = Parkinson's Progression Marker Initiative; FJMUUH-PD = Fujian Medical University Union Hospital Parkinson's Disease; FoG = freeze of gait; MDS-UPDRS = Movement Disorder Society Unified Parkinson's Disease Rating Scale; H&Y=Hoehn–Yahr; RBDSQ= REM sleep behavior disorder Screening Questionnaire; MoCA=Montreal Cognitive Assessment.

Categorical variables were compared using Chi-square tests, and the results were reported as counts and percentages (n (%)).

Normally distributed continuous variables were compared using independent *t* -tests, and the results were reported as means along with standard deviations (means ±standard deviations).

Non-normally distributed continuous variables were assessed using the Mann-Whitney *U*-test, and the results were reported as medians, along with interquartile ranges (medians [interquartile ranges]).

* Adjusted by false discovery rate.

Supplementary Table 2: Comparison of all cortical thickness variables according to FoG evolution.

variable	PPMI				FJMUUH-PD			
	FoG nonconverters n=60	FoG converters n=25	*P*-value	Adjusted *P*-value*	FoG nonconverters n=37	FoG converters n=18	*P*-value	Adjusted *P*-value*
lG_and_S_frontomargin	2.37±0.12	2.35±0.15	0.495	0.691	0.41±0.03	0.42±0.04	0.232	0.583
rG_and_S_frontomargin	2.38±0.13	2.41±0.15	0.472	0.685	0.43±0.03	0.43±0.04	0.341	0.647
lG_and_S_occipital_inf	2.38±0.15	2.33±0.23	0.191	0.436	0.57±0.1	0.61±0.09	0.950	0.997
rG_and_S_occipital_inf	2.5±0.17	2.42±0.21	0.07	0.264	0.56±0.09	0.57±0.11	0.005	0.080
lG_and_S_paracentral	2.13±0.18	1.98±0.34	0.042	0.19	0.44±0.04	0.42±0.05	0.426	0.742
rG_and_S_paracentral	2.14±0.17	2.05±0.32	0.226	0.464	0.41±0.04	0.39±0.05	0.051	0.293
lG_and_S_subcentral	2.59±0.13	2.48±0.2	0.025	0.166	0.37±0.03	0.35±0.02	0.069	0.343
rG_and_S_subcentral	2.58±0.12	2.52±0.2	0.205	0.447	0.37±0.04	0.35±0.02	0.035	0.276
lG_and_S_transv_frontopol	2.4±0.16	2.4±0.19	0.958	0.971	0.58±0.07	0.56±0.07	0.252	0.592
rG_and_S_transv_frontopol	2.36±0.16	2.39±0.16	0.413	0.658	0.54±0.04	0.51±0.03	0.580	0.818
lG_and_S_cingul-Ant	2.62±0.13	2.6±0.11	0.395	0.643	0.46±0.03	0.44±0.03	0.841	0.928
rG_and_S_cingul-Ant	2.6±0.1	2.57±0.16	0.419	0.66	0.45±0.04	0.44±0.04	0.148	0.457
lG_and_S_cingul-Mid-Ant	2.65±0.15	2.6±0.14	0.201	0.451	0.41±0.04	0.42±0.04	0.604	0.835
rG_and_S_cingul-Mid-Ant	2.66±0.12	2.58±0.15	0.012	0.13	0.4±0.04	0.4±0.05	0.590	0.823
lG_and_S_cingul-Mid-Post	2.55±0.12	2.49±0.13	0.032	0.175	0.42±0.04	0.44±0.05	0.286	0.623
rG_and_S_cingul-Mid-Post	2.58±0.11	2.5±0.18	0.044	0.192	0.43±0.05	0.46±0.08	0.304	0.625
lG_cingul-Post-dorsal	2.87±0.14	2.85±0.21	0.701	0.811	0.41±0.03	0.39±0.03	0.002	0.138
rG_cingul-Post-dorsal	2.85±0.17	2.73±0.25	0.023	0.161	0.39±0.03	0.38±0.03	0.004	0.110
lG_cingul-Post-ventral	2.5±0.24	2.36±0.23	0.018	0.145	0.48±0.03	0.5±0.05	0.621	0.844
rG_cingul-Post-ventral	2.53±0.24	2.56±0.25	0.704	0.808	0.46±0.04	0.46±0.04	0.938	0.998
lG_cuneus	1.89±0.12	1.87±0.4	0.654	0.781	0.74±0.12	0.61±0.12	0.080	0.347
rG_cuneus	1.95±0.11	1.85±0.21	0.027	0.166	0.74±0.13	0.62±0.12	0.315	0.630
lG_front_inf-Opercular	2.66±0.13	2.58±0.13	0.006	0.086	0.68±0.15	0.56±0.14	0.026	0.212
rG_front_inf-Opercular	2.67±0.12	2.66±0.21	0.734	0.811	0.68±0.14	0.56±0.14	0.976	0.996
lG_front_inf-Orbital	2.58±0.19	2.58±0.22	0.858	0.907	0.75±0.08	0.63±0.11	0.375	0.694
rG_front_inf-Orbital	2.53±0.19	2.48±0.21	0.312	0.557	0.72±0.09	0.63±0.11	0.526	0.787
lG_front_inf-Triangul	2.48±0.12	2.41±0.18	0.04	0.184	0.83±0.06	0.72±0.09	0.173	0.484
rG_front_inf-Triangul	2.47±0.11	2.52±0.17	0.145	0.369	0.79±0.08	0.67±0.1	0.475	0.764
lG_front_middle	2.5±0.1	2.48±0.15	0.492	0.694	0.82±0.1	0.7±0.12	0.103	0.411
rG_front_middle	2.47±0.1	2.49±0.16	0.534	0.694	0.76±0.13	0.63±0.13	0.449	0.730
lG_front_sup	2.67±0.11	2.6±0.23	0.142	0.374	0.78±0.11	0.66±0.13	0.269	0.602
rG_front_sup	2.66±0.11	2.64±0.16	0.589	0.732	0.79±0.13	0.66±0.12	0.079	0.355
lG_Ins_lg_and_S_cent_ins	3.29±0.28	3.14±0.37	0.05	0.207	0.77±0.13	0.65±0.14	0.000	0.009
rG_Ins_lg_and_S_cent_ins	3.23±0.31	3.11±0.36	0.17	0.421	0.77±0.13	0.65±0.14	0.482	0.760
lG_insular_short	3.83±0.25	3.68±0.43	0.052	0.208	0.68±0.12	0.55±0.13	0.085	0.358
rG_insular_short	3.72±0.34	3.63±0.38	0.289	0.555	0.68±0.13	0.55±0.13	0.189	0.500
lG_occipital_middle	2.43±0.13	2.4±0.12	0.456	0.681	0.75±0.1	0.66±0.11	0.836	0.930
rG_occipital_middle	2.46±0.1	2.4±0.13	0.035	0.173	0.77±0.11	0.68±0.12	0.430	0.741
lG_occipital_sup	2.1±0.11	2.09±0.27	0.737	0.808	0.69±0.12	0.57±0.12	0.773	0.894

rG_occipital_sup	2.16±0.11	2.13±0.18	0.321	0.566	0.7±0.14	0.59±0.12	0.556	0.799
lG_oc-temp_lat-fusifor	2.64±0.14	2.61±0.18	0.545	0.701	1.11±0.18	0.93±0.2	0.006	0.079
rG_oc-temp_lat-fusifor	2.63±0.18	2.52±0.18	0.012	0.139	1.12±0.2	0.96±0.19	0.036	0.265
lG_oc-temp_med-Lingual	2±0.13	1.99±0.29	0.709	0.802	1.2±0.31	1.02±0.25	0.222	0.567
rG_oc-temp_med-Lingual	2.08±0.13	2.01±0.15	0.023	0.168	1.2±0.29	0.99±0.26	0.521	0.795
lG_oc-temp_med-Parahip	2.76±0.19	2.74±0.19	0.555	0.708	1.15±0.14	0.96±0.17	0.246	0.587
rG_oc-temp_med-Parahip	3.04±0.21	2.96±0.36	0.201	0.444	1.07±0.14	0.94±0.17	0.169	0.489
lG_orbital	2.74±0.13	2.72±0.17	0.515	0.7	1.19±0.09	1.07±0.15	0.931	1.006
rG_orbital	2.8±0.13	2.73±0.19	0.124	0.376	1.15±0.13	1.01±0.19	0.003	0.122
lG_pariet_inf-Angular	2.45±0.11	2.46±0.12	0.92	0.959	1.44±0.22	1.25±0.22	0.003	0.127
rG_pariet_inf-Angular	2.5±0.11	2.46±0.16	0.173	0.42	1.31±0.22	1.08±0.25	0.004	0.121
lG_pariet_inf-Supramar	2.57±0.11	2.48±0.15	0.003	0.066	1.22±0.17	1.04±0.22	0.820	0.919
rG_pariet_inf-Supramar	2.61±0.09	2.56±0.17	0.133	0.377	1.22±0.21	1.04±0.2	0.549	0.797
lG_parietal_sup	2.27±0.11	2.29±0.16	0.641	0.771	1.15±0.2	1±0.21	0.777	0.891
rG_parietal_sup	2.28±0.11	2.3±0.19	0.524	0.693	1.15±0.21	0.96±0.19	0.037	0.248
lG_postcentral	2.06±0.13	1.98±0.16	0.017	0.154	1.02±0.17	0.86±0.21	0.686	0.867
rG_postcentral	2.03±0.14	1.98±0.26	0.29	0.549	1.03±0.2	0.87±0.2	0.692	0.868
lG_precentral	2.51±0.16	2.32±0.26	0.001	0.049	1.13±0.16	0.99±0.2	0.005	0.103
rG_precentral	2.43±0.21	2.37±0.23	0.181	0.42	1.13±0.17	1±0.19	0.173	0.491
lG_precuneus	2.44±0.12	2.49±0.22	0.267	0.542	1.09±0.19	0.94±0.2	0.697	0.867
rG_precuneus	2.45±0.1	2.43±0.15	0.366	0.608	1.09±0.2	0.96±0.2	0.493	0.768
lG_rectus	2.38±0.12	2.36±0.12	0.6	0.74	0.57±0.1	0.47±0.1	0.971	0.998
rG_rectus	2.41±0.14	2.42±0.17	0.899	0.944	0.56±0.1	0.48±0.11	0.143	0.450
lG_subcallosal	2.89±0.39	2.88±0.38	0.938	0.964	0.42±0.08	0.34±0.11	0.618	0.847
rG_subcallosal	2.9±0.3	2.92±0.37	0.775	0.837	0.43±0.07	0.35±0.1	0.253	0.586
lG_temp_sup-G_T_transv	2.36±0.19	2.21±0.23	0.005	0.095	0.55±0.06	0.49±0.11	0.525	0.794
rG_temp_sup-G_T_transv	2.36±0.19	2.26±0.28	0.118	0.364	0.56±0.07	0.5±0.1	0.439	0.739
lG_temp_sup-Lateral	2.91±0.15	2.76±0.19	<0.001	0.042	0.65±0.06	0.58±0.07	0.036	0.253
rG_temp_sup-Lateral	2.94±0.13	2.82±0.19	0.001	0.049	0.62±0.06	0.56±0.09	0.704	0.868
lG_temp_sup-Plan_polar	3.48±0.24	3.27±0.32	0.002	0.053	0.51±0.07	0.47±0.1	0.018	0.181
rG_temp_sup-Plan_polar	3.47±0.27	3.28±0.41	0.011	0.132	0.5±0.09	0.43±0.09	0.141	0.455
lG_temp_sup-Plan_tempo	2.51±0.12	2.42±0.14	0.002	0.06	0.57±0.08	0.5±0.1	0.515	0.794
rG_temp_sup-Plan_tempo	2.55±0.13	2.49±0.41	0.334	0.575	0.58±0.1	0.5±0.1	0.651	0.852
lG_temporal_inf	2.8±0.13	2.76±0.18	0.211	0.453	0.59±0.1	0.51±0.12	0.933	1.000
rG_temporal_inf	2.84±0.15	2.77±0.23	0.102	0.329	0.59±0.1	0.51±0.12	0.011	0.133
lG_temporal_middle	2.84±0.13	2.75±0.19	0.017	0.152	0.52±0.09	0.42±0.12	0.149	0.449
rG_temporal_middle	2.89±0.12	2.8±0.24	0.078	0.288	0.51±0.1	0.41±0.11	0.781	0.889
lLat_Fis-ant-Horizont	2.42±0.18	2.36±0.26	0.269	0.538	0.57±0.08	0.52±0.09	0.562	0.800
rLat_Fis-ant-Horizont	2.45±0.14	2.42±0.2	0.513	0.704	0.6±0.09	0.54±0.1	0.061	0.333
lLat_Fis-ant-Vertical	2.55±0.18	2.48±0.2	0.097	0.334	0.49±0.09	0.41±0.11	0.123	0.456
rLat_Fis-ant-Vertical	2.5±0.2	2.53±0.2	0.516	0.694	0.52±0.11	0.44±0.1	0.405	0.714
lLat_Fis-post	2.5±0.2	2.53±0.2	0.035	0.176	2.47±0.25	2.28±0.19	0.137	0.451
rLat_Fis-post	2.38±0.13	2.27±0.25	0.164	0.412	2.49±0.24	2.34±0.32	0.022	0.195
lPole_occipital	2.38±0.13	2.27±0.25	0.325	0.566	2.2±0.21	2.16±0.21	0.246	0.596

rPole_occipital	2.56±0.13	2.49±0.25	<0.001	0.021	2.38±0.23	2.28±0.2	0.478	0.761
lPole_temporal	2.56±0.13	2.49±0.25	0.031	0.182	1.91±0.24	2.04±0.22	0.741	0.892
rPole_temporal	2.02±0.11	1.96±0.27	0.034	0.178	1.92±0.27	1.99±0.24	0.261	0.594
lS_calcarine	2.02±0.11	1.96±0.27	0.447	0.675	2.22±0.27	2.35±0.2	0.660	0.850
rS_calcarine	2.05±0.12	1.93±0.18	0.031	0.176	2.14±0.28	2.26±0.23	0.747	0.878
lS_central	2.05±0.12	1.93±0.18	0.048	0.205	2.48±0.28	2.32±0.21	0.681	0.868
rS_central	3.42±0.22	3.31±0.25	0.139	0.373	2.53±0.3	2.34±0.26	0.432	0.734
lS_cingul-Marginalis	3.42±0.22	3.31±0.25	0.804	0.862	2.72±0.27	2.63±0.18	0.071	0.339
rS_cingul-Marginalis	3.4±0.23	3.22±0.53	0.294	0.543	2.68±0.25	2.58±0.19	0.021	0.195
lS_circular_insula_ant	3.4±0.23	3.22±0.53	0.1	0.33	2.68±0.26	2.61±0.17	0.005	0.093
rS_circular_insula_ant	1.99±0.16	1.96±0.29	0.082	0.298	2.62±0.24	2.61±0.18	0.051	0.303
lS_circular_insula_inf	1.99±0.16	1.96±0.29	0.006	0.089	2.45±0.21	2.46±0.16	0.108	0.419
rS_circular_insula_inf	2.01±0.15	1.93±0.15	0.006	0.095	2.45±0.25	2.48±0.16	0.328	0.638
lS_circular_insula_sup	2.01±0.15	1.93±0.15	0.037	0.175	2.56±0.44	2.7±0.33	0.005	0.086
rS_circular_insula_sup	1.81±0.15	1.71±0.22	0.098	0.329	2.56±0.39	2.68±0.18	0.161	0.476
lS_collat_transv_ant	1.81±0.15	1.71±0.22	0.062	0.24	2.14±0.37	2.2±0.34	0.742	0.879
rS_collat_transv_ant	1.78±0.17	1.7±0.28	0.144	0.374	2.15±0.27	2.21±0.28	0.318	0.627
lS_collat_transv_post	1.78±0.17	1.7±0.28	0.715	0.802	1.79±0.16	1.8±0.18	0.444	0.730
rS_collat_transv_post	2.3±0.11	2.31±0.17	0.181	0.424	1.78±0.19	1.8±0.13	0.739	0.897
lS_front_inf	2.3±0.11	2.31±0.17	0.949	0.968	2.24±0.25	2.34±0.21	0.960	0.993
rS_front_inf	2.35±0.11	2.32±0.13	0.44	0.678	2.27±0.25	2.36±0.26	0.297	0.627
lS_front_middle	2.35±0.11	2.32±0.13	0.925	0.957	2.43±0.23	2.47±0.24	0.136	0.457
rS_front_middle	3.12±0.19	2.98±0.38	0.281	0.546	2.44±0.27	2.41±0.21	0.065	0.344
lS_front_sup	3.12±0.19	2.98±0.38	0.763	0.831	2.35±0.2	2.32±0.17	0.923	1.004
rS_front_sup	3.13±0.23	3.03±0.27	0.561	0.709	2.31±0.24	2.29±0.15	0.442	0.735
lS_interm_prim-Jensen	3.13±0.23	3.03±0.27	0.126	0.372	2.3±0.2	2.35±0.09	0.131	0.462
rS_interm_prim-Jensen	3.04±0.22	2.88±0.25	0.665	0.787	2.33±0.16	2.34±0.14	0.233	0.574
lS_intrapariet_and_P_trans	3.04±0.22	2.88±0.25	0.111	0.351	2.46±0.25	2.52±0.13	0.542	0.794
rS_intrapariet_and_P_trans	2.99±0.22	2.84±0.24	0.708	0.805	2.43±0.25	2.48±0.18	0.900	0.987
lS_oc_middle_and_Lunatus	2.99±0.22	2.84±0.24	0.308	0.563	2.16±0.66	2.73±0.49	0.626	0.842
rS_oc_middle_and_Lunatus	2.83±0.12	2.73±0.21	0.216	0.456	2.64±0.45	2.87±0.33	0.180	0.486
lS_oc_sup_and_transversal	2.83±0.12	2.73±0.21	0.975	0.975	2.92±0.51	3.33±0.4	0.132	0.453
rS_oc_sup_and_transversal	2.93±0.14	2.87±0.15	0.812	0.864	3.08±0.47	3.38±0.38	0.535	0.792
lS_occipital_ant	2.93±0.14	2.87±0.15	0.385	0.633	2.43±0.16	2.4±0.18	0.017	0.176
rS_occipital_ant	2.75±0.15	2.68±0.16	0.137	0.382	2.52±0.15	2.39±0.16	0.946	1.000
lS_oc-temp_lat	2.75±0.15	2.68±0.16	0.484	0.696	2.03±0.23	2.03±0.14	0.761	0.887
rS_oc-temp_lat	2.74±0.16	2.68±0.19	0.343	0.577	1.99±0.2	2.1±0.15	0.123	0.446
lS_oc-temp_med_and_Lingual	2.74±0.16	2.68±0.19	0.275	0.544	2.05±0.28	2.19±0.27	0.179	0.491
rS_oc-temp_med_and_Lingual	2.1±0.15	2.09±0.18	0.457	0.676	2.06±0.27	2.18±0.28	0.405	0.722
lS_orbital_lateral	2.1±0.15	2.09±0.18	0.469	0.687	1.84±0.13	1.8±0.16	0.202	0.525
rS_orbital_lateral	2.11±0.15	2.06±0.17	0.53	0.694	1.9±0.14	1.86±0.14	0.040	0.259
lS_orbital_med-olfact	2.11±0.15	2.06±0.17	0.974	0.98	2.24±0.3	2.44±0.27	0.984	0.991

rS_orbital_med-olfact	2.34±0.11	2.34±0.14	0.226	0.47	2.38±0.27	2.62±0.32	0.299	0.624
lS_orbital-H_Shaped	2.34±0.11	2.34±0.14	0.667	0.783	2.41±0.17	2.41±0.15	0.391	0.706
rS_orbital-H_Shaped	2.34±0.11	2.37±0.16	0.632	0.773	2.4±0.15	2.46±0.16	0.328	0.631
lS_parieto_occipital	2.34±0.11	2.37±0.16	0.681	0.794	2.42±0.18	2.38±0.12	0.646	0.862
rS_parieto_occipital	2.34±0.11	2.33±0.16	0.025	0.159	2.4±0.19	2.38±0.12	0.276	0.610
lS_pericallosal	2.34±0.11	2.33±0.16	0.018	0.143	2.35±0.19	2.38±0.1	0.012	0.137
rS_pericallosal	2.32±0.11	2.35±0.13	0.337	0.574	2.33±0.18	2.38±0.14	0.649	0.858
lS_postcentral	2.32±0.11	2.35±0.13	0.64	0.776	2.12±0.18	2.18±0.15	0.050	0.311
rS_postcentral	2.48±0.1	2.47±0.17	0.292	0.546	2.1±0.21	2.14±0.15	0.121	0.457
lS_precentral-inf-part	2.48±0.1	2.47±0.17	0.09	0.319	1.93±0.19	1.97±0.19	0.995	0.995
rS_precentral-inf-part	2.47±0.11	2.49±0.14	0.588	0.737	1.88±0.2	1.9±0.22	0.361	0.677
lS_precentral-sup-part	2.47±0.11	2.49±0.14	0.438	0.682	2.25±0.28	2.38±0.26	0.390	0.713
rS_precentral-sup-part	2.43±0.18	2.37±0.16	0.52	0.693	2.25±0.28	2.25±0.29	0.979	0.992
lS_suborbital	2.43±0.18	2.37±0.16	0.44	0.672	2.27±0.24	2.32±0.17	0.959	1.000
rS_suborbital	2.38±0.14	2.37±0.21	0.312	0.563	2.23±0.22	2.29±0.16	0.313	0.634
lS_subparietal	2.38±0.14	2.37±0.21	0.403	0.649	2.25±0.19	2.19±0.19	0.293	0.628
rS_subparietal	2.19±0.09	2.23±0.13	0.733	0.816	2.27±0.2	2.19±0.15	0.785	0.887
lS_temporal_inf	2.19±0.09	2.23±0.13	0.016	0.158	2.43±0.54	2.68±0.29	0.103	0.421
rS_temporal_inf	2.2±0.1	2.21±0.17	0.131	0.379	2.26±0.48	2.5±0.33	0.067	0.340
lS_temporal_sup	2.2±0.1	2.21±0.17	0.488	0.694	1.72±0.28	2.05±0.25	0.741	0.885
rS_temporal_sup	2.16±0.1	2.13±0.17	0.138	0.379	1.82±0.29	1.81±0.46	0.078	0.360
lS_temporal_transverse	2.16±0.1	2.13±0.17	0.176	0.421	2.49±0.24	2.54±0.19	0.708	0.867
rS_temporal_transverse	2.23±0.13	2.2±0.13	0.503	0.696	2.46±0.32	2.53±0.23	0.652	0.846

PPMI = Parkinson’s Progression Marker Initiative; FJMUUH-PD = Fujian Medical University Union Hospital Parkinson’s Disease; FoG = freeze of gait

Normally distributed continuous variables were compared using independent *t* -tests, and the results were reported as means along with standard deviations (means ±standard deviations).

* Adjusted by false discovery rate.

Supplementary Table 3: Comparison of all white matter fiber variables according to FoG evolution.

variable	PPMI				FJMUUH-PD			
	FoG nonconverters n=60	FoG converters n=25	*P*-value	Adjusted *P*-value*	FoG nonconverters n=37	FoG converters n=18	*P*-value	Adjusted *P*-value*
FA-Left Thalamic Radiation	0.47±0.03	0.45±0.04	0.03	0.185	2.56±0.42	2.78±0.44	0.777	0.797
FA-Right Thalamic Radiation	0.48±0.03	0.46±0.02	0.012	0.16	2.61±0.43	2.79±0.37	0.676	0.722
FA-Left Corticospinal	0.65±0.04	0.64±0.04	0.389	0.598	2.18±0.2	2.25±0.13	0.128	0.149
FA-Right Corticospinal	0.62±0.03	0.61±0.03	0.395	0.585	2.12±0.24	2.2±0.24	0.680	0.716
FA-Left Cingulum Cingulate	0.53±0.05	0.51±0.06	0.384	0.602	2.55±0.22	2.54±0.17	0.102	0.120
FA-Right Cingulum Cingulate	0.49±0.05	0.5±0.04	0.376	0.602	2.58±0.18	2.55±0.17	0.246	0.277
FA-Left Cingulum Hippocampus	0.43±0.04	0.43±0.05	0.908	0.956	2.58±0.23	2.63±0.13	0.057	0.072
FA-Right Cingulum Hippocampus	0.41±0.04	0.43±0.05	0.402	0.585	2.66±0.22	2.65±0.14	0.013	0.021
FA-Callosum Forceps Major	0.61±0.06	0.58±0.16	0.276	0.566	2.29±0.24	2.31±0.17	0.493	0.540
FA-Callosum Forceps Minor	0.53±0.03	0.5±0.07	0.154	0.474	2.31±0.24	2.27±0.15	0.043	0.057
FA-Left IFOF	0.49±0.04	0.48±0.04	0.453	0.594	2.22±0.3	2.27±0.33	0.046	0.060
FA-Right IFOF	0.49±0.03	0.47±0.04	0.355	0.604	2.21±0.29	2.32±0.28	0.774	0.804
FA-Left ILF	0.45±0.04	0.44±0.05	0.224	0.543	1.82±0.34	2.08±0.25	0.833	0.844
FA-Right ILF	0.43±0.03	0.42±0.04	0.48	0.61	2.02±0.37	2.18±0.32	0.504	0.545
FA-Left SLF	0.47±0.05	0.48±0.03	0.562	0.671	1.99±0.19	1.87±0.15	0.054	0.069
FA-Right SLF	0.48±0.04	0.5±0.05	0.423	0.583	1.94±0.19	1.91±0.12	0.152	0.174
FA-Left Uncinate	0.43±0.04	0.4±0.03	0.05	0.25	2.73±0.38	2.87±0.41	0.030	0.041
FA-Right Uncinate	0.41±0.04	0.39±0.04	0.135	0.45	2.68±0.32	2.91±0.33	0.064	0.078
FA-Left Arcuate	0.52±0.03	0.51±0.03	0.635	0.726	1.91±0.16	1.87±0.19	0.256	0.285
FA-Right Arcuate	0.48±0.04	0.46±0.05	0.297	0.528	1.86±0.17	1.86±0.21	0.924	0.924
**MD-Left Thalamic Radiation**	**0.72±0.03**	**0.77±0.05**	**<0.001**	**0.015**	1.68±0.18	1.74±0.15	0.000	0.004
MD-Right Thalamic Radiation	0.73±0.03	0.76±0.04	0.004	0.08	1.71±0.2	1.69±0.19	0.002	0.006
MD-Left Corticospinal	0.68±0.03	0.71±0.03	0.012	0.137	2.13±0.21	2.21±0.17	0.004	0.010
MD-Right Corticospinal	0.73±0.03	0.74±0.04	0.218	0.563	2.14±0.22	2.22±0.13	0.004	0.010

MD-Left Cingulum Cingulate	0.72±0.03	0.74±0.06	0.272	0.588	2.72±0.28	2.72±0.19	0.000	0.001
MD-Right Cingulum Cingulate	0.72±0.04	0.72±0.05	0.959	0.971	2.71±0.28	2.82±0.28	0.001	0.007
MD-Left Cingulum Hippocampus	0.72±0.06	0.72±0.05	0.916	0.952	2.06±0.45	2.44±0.39	0.000	0.000
MD-Right Cingulum Hippocampus	0.77±0.06	0.77±0.08	0.82	0.886	2.3±0.41	2.4±0.36	0.000	0.000
MD-Callosum Forceps Major	0.83±0.1	0.78±0.1	0.133	0.463	2.18±0.37	2.49±0.28	0.000	0.003
MD-Callosum Forceps Minor	0.78±0.03	0.81±0.06	0.014	0.124	2.44±0.3	2.56±0.36	0.001	0.005
MD-Left IFOF	0.78±0.03	0.8±0.05	0.164	0.486	2.25±0.23	2.44±0.22	0.000	0.004
MD-Right IFOF	0.79±0.04	0.82±0.06	0.269	0.598	2.31±0.23	2.45±0.18	0.001	0.007
MD-Left ILF	0.76±0.04	0.78±0.07	0.314	0.546	1.83±0.21	1.85±0.23	0.003	0.009
MD-Right ILF	0.8±0.04	0.82±0.05	0.264	0.603	1.85±0.2	1.85±0.2	0.004	0.009
MD-Left SLF	0.68±0.04	0.7±0.06	0.216	0.576	2.26±0.18	2.24±0.15	0.000	0.004
MD-Right SLF	0.71±0.04	0.72±0.06	0.403	0.576	2.3±0.15	2.25±0.15	0.001	0.006
MD-Left Uncinate	0.76±0.04	0.79±0.05	0.031	0.177	2.21±0.22	2.22±0.15	0.002	0.007
MD-Right Uncinate	0.79±0.05	0.83±0.05	0.013	0.13	2.26±0.17	2.22±0.13	0.008	0.015
MD-Left Arcuate	0.69±0.03	0.71±0.06	0.359	0.598	2.24±0.21	2.36±0.14	0.001	0.006
MD-Right Arcuate	0.7±0.03	0.72±0.06	0.287	0.534	2.26±0.22	2.31±0.13	0.005	0.010
AD-Left Thalamic Radiation	1.13±0.05	1.17±0.06	0.008	0.128	2.4±0.34	2.37±0.18	0.002	0.006
AD-Right Thalamic Radiation	1.15±0.05	1.18±0.05	0.148	0.474	2.25±0.19	2.27±0.2	0.006	0.013
AD-Left Corticospinal	1.28±0.05	1.32±0.05	0.015	0.12	2.08±0.19	2.1±0.13	0.040	0.054
AD-Right Corticospinal	1.33±0.04	1.34±0.05	0.521	0.651	2.05±0.18	2.1±0.15	0.012	0.019
AD-Left Cingulum Cingulate	1.19±0.06	1.2±0.08	0.547	0.663	2.28±0.18	2.12±0.22	0.000	0.002
AD-Right Cingulum Cingulate	1.15±0.06	1.16±0.07	0.406	0.57	2.32±0.17	2.19±0.19	0.004	0.010
AD-Left Cingulum Hippocampus	1.08±0.07	1.08±0.06	0.851	0.908	2.16±0.22	2.08±0.15	0.000	0.003
AD-Right Cingulum Hippocampus	1.14±0.08	1.16±0.08	0.543	0.668	2.13±0.21	2.16±0.11	0.002	0.006
AD-Callosum Forceps Major	1.52±0.13	1.41±0.28	0.063	0.296	2.25±0.22	2.28±0.16	0.003	0.009
AD-Callosum Forceps Minor	1.32±0.04	1.32±0.03	0.791	0.867	2.42±0.27	2.28±0.23	0.001	0.006
AD-Left IFOF	1.24±0.05	1.25±0.06	0.432	0.586	2.18±0.25	2.23±0.23	0.001	0.006
AD-Right IFOF	1.26±0.06	1.28±0.06	0.361	0.589	2.16±0.18	2.25±0.26	0.004	0.010

AD-Left ILF	1.16±0.05	1.17±0.08	0.394	0.595	2.11±0.24	2.11±0.17	0.013	0.021
AD-Right ILF	1.2±0.07	1.21±0.04	0.293	0.533	2.08±0.2	2.08±0.18	0.003	0.008
AD-Left SLF	1.06±0.07	1.09±0.06	0.1	0.4	2.36±0.32	2.24±0.19	0.005	0.010
AD-Right SLF	1.11±0.05	1.15±0.06	0.037	0.197	2.37±0.33	2.29±0.22	0.007	0.014
AD-Left Uncinate	1.14±0.05	1.16±0.06	0.221	0.553	2.18±0.27	2.14±0.2	0.009	0.015
AD-Right Uncinate	1.16±0.05	1.19±0.04	0.077	0.342	2.08±0.24	2.14±0.23	0.016	0.025
AD-Left Arcuate	1.12±0.04	1.15±0.07	0.204	0.563	2.34±0.2	2.28±0.14	0.006	0.012
AD-Right Arcuate	1.1±0.05	1.12±0.07	0.449	0.599	2.33±0.18	2.33±0.16	0.029	0.041
**RD-Left Thalamic Radiation**	**0.52±0.04**	**0.57±0.05**	**<0.001**	**0.009**	1.95±0.23	2±0.22	0.002	0.006
**RD-Right Thalamic Radiation**	**0.51±0.03**	**0.55±0.04**	**0.001**	**0.027**	2.04±0.19	2.04±0.24	0.010	0.017
RD-Left Corticospinal	0.38±0.04	0.4±0.04	0.082	0.345	1.92±0.32	1.84±0.3	0.002	0.006
RD-Right Corticospinal	0.43±0.04	0.44±0.04	0.237	0.558	1.91±0.39	1.81±0.22	0.002	0.006
RD-Left Cingulum Cingulate	0.48±0.05	0.51±0.07	0.129	0.469	2±0.16	2.09±0.12	0.008	0.015
RD-Right Cingulum Cingulate	0.51±0.05	0.5±0.04	0.642	0.723	1.99±0.18	2.04±0.14	0.030	0.041
RD-Left Cingulum Hippocampus	0.54±0.06	0.54±0.06	0.966	0.966	2.3±0.19	2.32±0.16	0.000	0.003
RD-Right Cingulum Hippocampus	0.58±0.05	0.58±0.08	0.919	0.943	2.34±0.2	2.36±0.16	0.002	0.006
RD-Callosum Forceps Major	0.49±0.1	0.47±0.08	0.457	0.59	2.17±0.29	2.35±0.17	0.081	0.096
RD-Callosum Forceps Minor	0.51±0.04	0.56±0.09	0.122	0.465	2.16±0.28	2.29±0.19	0.021	0.030
RD-Left IFOF	0.55±0.04	0.57±0.05	0.193	0.551	2.63±0.31	2.5±0.33	0.010	0.017
RD-Right IFOF	0.56±0.04	0.58±0.06	0.28	0.56	2.58±0.37	2.4±0.29	0.007	0.013
RD-Left ILF	0.56±0.05	0.59±0.08	0.272	0.573	2.37±0.24	2.33±0.22	0.017	0.025
RD-Right ILF	0.6±0.04	0.62±0.06	0.283	0.539	2.41±0.19	2.39±0.21	0.011	0.019
RD-Left SLF	0.5±0.04	0.51±0.05	0.608	0.705	2.38±0.19	2.4±0.11	0.001	0.006
RD-Right SLF	0.5±0.04	0.51±0.06	0.779	0.866	2.43±0.2	2.34±0.13	0.001	0.006
RD-Left Uncinate	0.57±0.05	0.61±0.05	0.022	0.147	2.34±0.14	2.33±0.12	0.024	0.034
RD-Right Uncinate	0.6±0.05	0.64±0.06	0.016	0.116	2.36±0.17	2.35±0.11	0.063	0.078
RD-Left Arcuate	0.47±0.03	0.48±0.05	0.575	0.676	1.85±0.23	2.03±0.24	0.007	0.013
RD-Right Arcuate	0.5±0.04	0.53±0.06	0.28	0.546	1.84±0.28	1.88±0.32	0.016	0.024

PPMI = Parkinson's Progression Marker Initiative; FJMUUH-PD = Fujian Medical University Union Hospital Parkinson's Disease; FoG = freeze of gait; FA= fractional anisotropy; MD=mean diffusivity; AD=axial diffusion; RD=radial diffusion

Normally distributed continuous variables were compared using independent *t* -tests, and the results were reported as means along with standard deviations (means ±standard deviations).

* Adjusted by false discovery rate.

Supplementary Table 4. Performances of machine learning models to predict in the test set of the PPMI cohort.

Model	Over-sampling Method	Clinical	Cortical thickness	White fiber tract	Clinical & Cortical thickness	Clinical &White fiber tract	Clinical & Cortical thickness &White fiber tract
SVM	SMOTE	0.66(0.46-0.93)	0.56(0.45-0.67)	0.59(0.4-0.85)	0.73(0.5-0.94)	0.62(0.43-0.82)	0.83(0.61-0.97)
(linear)	ROSE	0.68(0.46-0.93)	0.72(0.64-0.79)	0.57(0.39-0.84)	0.73(0.53-0.95)	0.64(0.47-0.84)	0.84(0.6-0.98)
SVM	SMOTE	0.61(0.44-0.92)	0.59(0.44-0.66)	0.59(0.36-0.78)	0.76(0.48-0.98)	0.62(0.39-0.8)	0.83(0.65-0.99)
(polynomial)	ROSE	0.63(0.47-0.94)	0.77(0.71-0.81)	0.61(0.37-0.87)	0.74(0.43-0.97)	0.63(0.4-0.87)	0.86(0.53-0.99)
SVM	SMOTE	0.63(0.46-0.9)	0.63(0.51-0.67)	0.86(0.51-1)	0.78(0.58-0.95)	0.82(0.58-1)	0.87(0.66-0.99)
(radial)	ROSE	0.64(0.48-0.97)	0.55(0.43-0.62)	0.83(0.58-1)	0.81(0.58-0.98)	0.78(0.53-0.94)	0.88(0.72-1)
SVM	SMOTE	0.66(0.49-0.86)	0.64(0.6-0.67)	0.61(0.37-0.81)	0.71(0.48-0.92)	0.6(0.42-0.84)	0.76(0.5-0.97)
(sigmoid)	ROSE	0.69(0.47-0.91)	0.55(0.45-0.63)	0.57(0.4-0.78)	0.66(0.48-0.93)	0.59(0.43-0.79)	0.67(0.46-0.83)

The results were reported as Median AUC (lower, upper 2.5th %tile).

ROSE = random over-sampling examples; SMOTE = synthetic minority over-sampling technique; SVM = support vector machine.

Supplementary Table 5: Performances of machine learning models to predict in the test set of the FJMUUH-PD cohort.

Model	Over-sampling Method	Clinical	Cortical thickness	White fiber tract	Clinical & Cortical thickness	Clinical &White fiber tract	Clinical & Cortical thickness &White fiber tract
SVM	SMOTE	0.58(0.49-0.62)	0.72(0.5-0.92)	0.79(0.71-0.84)	0.63(0.52-0.67)	0.77(0.67-0.81)	0.8(0.71-0.85)
(linear)	ROSE	0.58(0.51-0.63)	0.74(0.55-0.96)	0.79(0.76-0.82)	0.63(0.57-0.67)	0.76(0.74-0.78)	0.81(0.77-0.85)
SVM	SMOTE	0.65(0.46-0.73)	0.73(0.49-0.92)	0.77(0.67-0.82)	0.75(0.63-0.85)	0.78(0.68-0.85)	0.82(0.72-0.87)
(polynomial)	ROSE	0.64(0.44-0.73)	0.81(0.6-0.97)	0.79(0.69-0.83)	0.78(0.67-0.86)	0.79(0.69-0.85)	0.84(0.78-0.88)
SVM	SMOTE	0.83(0.71-0.9)	0.72(0.47-0.96)	0.87(0.82-0.92)	0.81(0.7-0.88)	0.86(0.79-0.89)	0.88(0.83-0.93)
(radial)	ROSE	0.84(0.77-0.89)	0.72(0.44-0.91)	0.87(0.83-0.92)	0.84(0.76-0.9)	0.86(0.81-0.9)	0.91(0.86-0.95)
SVM	SMOTE	0.55(0.46-0.65)	0.74(0.52-0.93)	0.76(0.7-0.8)	0.57(0.45-0.64)	0.73(0.66-0.78)	0.77(0.64-0.82)
(sigmoid)	ROSE	0.62(0.42-0.67)	0.71(0.5-0.93)	0.71(0.59-0.79)	0.53(0.45-0.61)	0.66(0.56-0.73)	0.66(0.58-0.73)

The results were reported as Median AUC (lower, upper 2.5th %tile).

ROSE = random over-sampling examples; SMOTE = synthetic minority over-sampling technique; SVM = support vector machine.

Supplementary Table 6. Pair-wise comparisons of model performances per iteration according to feature combination PPMI.

Model	Over-sampling Method	Clinical& Cortical thickness &White fiber tract			P-value	Cortical thickness vs. Clinical & Cortical thickness &White fiber tract			P-value
		<0	0	>0		<0	0	>0	
SVM	SMOTE	86	0	14	<0.001	97	0	3	<0.001
(linear)	ROSE	90	2	8	<0.001	85	0	15	<0.001
SVM	SMOTE	85	5	10	<0.001	98	0	2	<0.001
(polynomial)	ROSE	90	1	9	<0.001	81	0	19	<0.001
SVM	SMOTE	93	0	7	<0.001	99	0	1	<0.001
(radial)	ROSE	93	0	7	<0.001	100	0	0	<0.001
SVM	SMOTE	71	4	25	<0.001	83	0	17	<0.001
(sigmoid)	ROSE	44	3	53	0.549	86	0	14	<0.001

Model	Over-sampling Method	White fiber tract vs. Clinical & Cortical thickness &White fiber tract			P-value	Clinical & Cortical thickness vs. Clinical & Cortical thickness &White fiber tract			P-value
		<0	0	>0		<0	0	>0	
SVM	SMOTE	96	0	4	<0.001	77	2	21	<0.001
(linear)	ROSE	95	2	3	<0.001	78	4	18	<0.001
SVM	SMOTE	97	0	3	<0.001	68	4	28	<0.001
(polynomial)	ROSE	93	0	7	<0.001	76	1	23	<0.001
SVM	SMOTE	43	5	52	0.557	69	6	25	<0.001
(radial)	ROSE	60	2	38	0.002	64	4	32	<0.001
SVM	SMOTE	81	3	16	<0.001	61	4	35	0.003
(sigmoid)	ROSE	73	1	26	<0.001	47	4	49	0.741

Model	Over-sampling Method	Clinical &White fiber tract vs. Clinical & Cortical thickness &White fiber tract			P-value
		<0	0	>0	
SVM	SMOTE	96	1	3	<0.001
(linear)	ROSE	95	2	3	<0.001
SVM	SMOTE	94	2	4	<0.001
(polynomial)	ROSE	95	1	4	<0.001
SVM	SMOTE	64	2	34	<0.001
(radial)	ROSE	81	3	16	<0.001
SVM	SMOTE	86	2	12	<0.001
(sigmoid)	ROSE	77	2	21	<0.001

Data are numbers of iterations which showed different or same performances between paired models.

Data are compared using independent t -tests.

Supplementary Table 7. Pair-wise comparisons of model performances per iteration according to feature combination FJMUUH-PD

Model	Over-sampling Method	Clinical& Cortical thickness &White fiber tract			P-value	Cortical thickness vs. Clinical & Cortical thickness &White fiber tract			P-value
		<0	0	>0		<0	0	>0	
SVM	SMOTE	100	0	0	<0.001	72	0	28	<0.001
(linear)	ROSE	100	0	0	<0.001	74	1	25	<0.001
SVM	SMOTE	100	0	0	<0.001	69	0	31	<0.001
(polynomial)	ROSE	100	0	0	<0.001	59	0	41	0.002
SVM	SMOTE	89	1	10	<0.001	90	0	10	<0.001
(radial)	ROSE	99	0	1	<0.001	98	0	2	<0.001
SVM	SMOTE	99	0	1	<0.001	49	0	51	0.079
(sigmoid)	ROSE	81	0	19	<0.001	32	0	68	<0.001
Model	Over-sampling Method	White fiber tract vs. Clinical & Cortical thickness &White fiber tract			P-value	Clinical & Cortical thickness vs. Clinical & Cortical thickness &White fiber tract			P-value
		<0	0	>0		<0	0	>0	
SVM	SMOTE	56	1	43	0.18	100	0	0	<0.001
(linear)	ROSE	84	0	16	<0.001	100	0	0	<0.001
SVM	SMOTE	90	1	9	<0.001	88	0	12	<0.001
(polynomial)	ROSE	92	0	8	<0.001	88	0	12	<0.001
SVM	SMOTE	70	4	26	<0.001	97	1	2	<0.001
(radial)	ROSE	88	1	11	<0.001	96	0	4	<0.001
SVM	SMOTE	56	1	43	0.291	100	0	0	<0.001
(sigmoid)	ROSE	12	0	88	<0.001	99	0	1	<0.001
Model	Over-sampling Method	Clinical &White fiber tract vs. Clinical & Cortical thickness &White fiber tract			P-value				
		<0	0	>0					
SVM	SMOTE	79	1	20	<0.001				
(linear)	ROSE	99	0	1	<0.001				
SVM	SMOTE	79	3	18	<0.001				
(polynomial)	ROSE	91	2	7	<0.001				
SVM	SMOTE	87	3	10	<0.001				
(radial)	ROSE	100	0	0	<0.001				
SVM	SMOTE	81	2	17	<0.001				
(sigmoid)	ROSE	49	0	51	0.677				

Data are numbers of iterations which showed different or same performances between paired models.

Data are compared using independent t -tests.

Supplementary Table 8: List of clinical, cortical thickness and white matter fiber variables used as features

	Features
Clinical variables (n = 19)	Age at baseline(y), Sex (%), Years of education, H&Y (%), MDS-UPDRS-I scores, MDS-UPDRS-II scores, MDS-UPDRS-III scores, UPDRS-I scores, UPDRS-II scores, UPDRS-III scores, RBDSQ, MoCA-Visuoconstructional Skills, MoCA-Naming, MoCA-Delayed Recall, MoCA-Attention, MoCA-Verbal Fluency, MoCA-Abstraction, MoCA-Orientation, MoCA-Total
Cortical thickness variables (*n* = 148)	lG_and_S_frontomargin, rG_and_S_frontomargin, lG_and_S_occipital_inf, rG_and_S_occipital_inf, lG_and_S_paracentral, rG_and_S_paracentral, lG_and_S_subcentral, rG_and_S_subcentral, lG_and_S_transv_frontopol, rG_and_S_transv_frontopol, lG_and_S_cingul-Ant, rG_and_S_cingul-Ant, lG_and_S_cingul-Mid-Ant, rG_and_S_cingul-Mid-Ant, lG_and_S_cingul-Mid-Post, rG_and_S_cingul-Mid-Post, lG_cingul-Post-dorsal, rG_cingul-Post-dorsal, lG_cingul-Post-ventral, rG_cingul-Post-ventral, lG_cuneus, rG_cuneus, lG_front_inf-Opercular, rG_front_inf-Opercular, lG_front_inf-Orbital, rG_front_inf-Orbital, lG_front_inf-Triangul, rG_front_inf-Triangul, lG_front_middle, rG_front_middle, lG_front_sup, rG_front_sup, lG_Ins_lg_and_S_cent_ins, rG_Ins_lg_and_S_cent_ins, lG_insular_short, rG_insular_short, lG_occipital_middle, rG_occipital_middle, lG_occipital_sup, rG_occipital_sup, lG_oc-temp_lat-fusifor, rG_oc-temp_lat-fusifor, lG_oc-temp_med-Lingual, rG_oc-temp_med-Lingual, lG_oc-temp_med-Parahip, rG_oc-temp_med-Parahip, lG_orbital, rG_orbital, lG_pariet_inf-Angular, rG_pariet_inf-Angular, lG_pariet_inf-Supramar, rG_pariet_inf-Supramar, lG_parietal_sup, rG_parietal_sup, lG_postcentral, rG_postcentral, lG_precentral, rG_precentral, lG_precuneus, rG_precuneus, lG_rectus, rG_rectus, lG_subcallosal, rG_subcallosal, lG_temp_sup-G_T_transv, rG_temp_sup-G_T_transv, lG_temp_sup-Lateral, rG_temp_sup-Lateral, lG_temp_sup-Plan_polar, rG_temp_sup-Plan_polar, lG_temp_sup-Plan_tempo, rG_temp_sup-Plan_tempo, lG_temporal_inf, rG_temporal_inf, lG_temporal_middle, rG_temporal_middle, lLat_Fis-ant-Horizont, rLat_Fis-ant-Horizont, lLat_Fis-ant-Vertical, rLat_Fis-ant-Vertical, lLat_Fis-post, rLat_Fis-post, lPole_occipital, rPole_occipital, lPole_temporal, rPole_temporal, lS_calcarine, rS_calcarine, lS_central, rS_central, lS_cingul-Marginalis, rS_cingul-Marginalis, lS_circular_insula_ant, rS_circular_insula_ant, lS_circular_insula_inf, rS_circular_insula_inf, lS_circular_insula_sup, rS_circular_insula_sup, lS_collat_transv_ant, rS_collat_transv_ant, lS_collat_transv_post, rS_collat_transv_post, lS_front_inf, rS_front_inf, lS_front_middle, rS_front_middle, lS_front_sup, rS_front_sup, lS_interm_prim-Jensen, rS_interm_prim-Jensen, lS_intrapariet_and_P_trans, rS_intrapariet_and_P_trans, lS_oc_middle_and_Lunatus, rS_oc_middle_and_Lunatus, lS_oc_sup_and_transversal, rS_oc_sup_and_transversal, lS_occipital_ant, rS_occipital_ant, lS_oc-temp_lat, rS_oc-temp_lat, lS_oc-temp_med_and_Lingual, rS_oc-temp_med_and_Lingual, lS_orbital_lateral, rS_orbital_lateral, lS_orbital_med-olfact, rS_orbital_med-olfact, lS_orbital-H_Shaped, rS_orbital-H_Shaped, lS_parieto_occipital, rS_parieto_occipital, lS_pericallosal, rS_pericallosal, lS_postcentral, rS_postcentral, lS_precentral-inf-part, rS_precentral-inf-part, lS_precentral-sup-part, rS_precentral-sup-part, lS_suborbital, rS_suborbital, lS_subparietal, rS_subparietal, lS_temporal_inf, rS_temporal_inf, lS_temporal_sup, rS_temporal_sup, lS_temporal_transverse, rS_temporal_transverse
White matter fiber variables (n=80)	FA-Left-Thalamic-Radiation, FA-Right-Thalamic-Radiation, FA-Left-Corticospinal, FA-Right-Corticospinal, FA-Left-Cingulum-Cingulate, FA-Right-Cingulum-Cingulate, FA-Left-Cingulum-Hippocampus, FA-Right-Cingulum-Hippocampus, FA-Callosum-Forceps-Major, FA-Callosum-Forceps-Minor, FA-Left-IFOF, FA-Right-IFOF, FA-Left-ILF, FA-Right-ILF, FA-Left-SLF, FA-Right-SLF, FA-Left-Uncinate, FA-Right-Uncinate, FA-Left-Arcuate, FA-Right-Arcuate, MD-Left-Thalamic-Radiation, MD-Right-Thalamic-Radiation, MD-Left-Corticospinal, MD-Right-Corticospinal, MD-Left-Cingulum-Cingulate, MD-Right-Cingulum-Cingulate, MD-Left-Cingulum-Hippocampus, MD-Right-Cingulum-Hippocampus, MD-Callosum-Forceps-Major, MD-Callosum-Forceps-Minor, MD-Left-IFOF, MD-Right-IFOF, MD-Left-ILF, MD-Right-ILF, MD-Left-SLF, MD-Right-SLF, MD-Left-Uncinate, MD-Right-Uncinate, MD-Left-Arcuate, MD-Right-Arcuate, AD-Left-Thalamic-Radiation, AD-Right-Thalamic-Radiation, AD-

	Left-Corticospinal, AD-Right-Corticospinal, AD-Left-Cingulum-Cingulate, AD-Right-Cingulum-Cingulate, AD-Left-Cingulum-Hippocampus, AD-Right-Cingulum-Hippocampus, AD-Callosum-Forceps-Major, AD-Callosum-Forceps-Minor, AD-Left-IFOF, AD-Right-IFOF, AD-Left-ILF, AD-Right-ILF, AD-Left-SLF, AD-Right-SLF, AD-Left-Uncinate, AD-Right-Uncinate, AD-Left-Arcuate, AD-Right-Arcuate, RD-Left-Thalamic-Radiation, RD-Right-Thalamic-Radiation, RD-Left-Corticospinal, RD-Right-Corticospinal, RD-Left-Cingulum-Cingulate, RD-Right-Cingulum-Cingulate, RD-Left-Cingulum-Hippocampus, RD-Right-Cingulum-Hippocampus, RD-Callosum-Forceps-Major, RD-Callosum-Forceps-Minor, RD-Left-IFOF, RD-Right-IFOF, RD-Left-ILF, RD-Right-ILF, RD-Left-SLF, RD-Right-SLF, RD-Left-Uncinate, RD-Right-Uncinate, RD-Left-Arcuate, RD-Right-Arcuate

H&Y=Hoehn–Yahr; MDS-UPDRS=Movement Disorder Society Unified Parkinson's Disease Rating Scale; MoCA=Montreal Cognitive Assessment; FA= fractional anisotropy; MD=mean diffusivity; AD=axial diffusion; RD=radial diffusion
