# Supplementary material for: Cortical thickness and white matter microstructure predict freezing of gait development in Parkinson’s disease
Source: NPJ Parkinsons Dis. 2024 Jan 9;10:16. doi: 10.1038/s41531-024-00629-x (PMC10776850; doi:10.1038/s41531-024-00629-x)
Supplement: Supplementary file 2 — REPORTING SUMMARY [file 41531_2024_629_MOESM2_ESM.pdf]

nature portfolio

Corresponding author(s): Guoen Cai

Last updated by author(s): Nov 14, 2023

Reporting Summary

Nature Portfolio wishes to improve the reproducibility of the work that we publish. This form provides structure for consistency and transparency in reporting. For further information on Nature Portfolio policies, see our Editorial Policies and the Editorial Policy Checklist.

Statistics

For all statistical analyses, confirm that the following items are present in the figure legend, table legend, main text, or Methods section.

n/a	Confirmed	
☐	☒	The exact sample size (n) for each experimental group/condition, given as a discrete number and unit of measurement
☐	☒	A statement on whether measurements were taken from distinct samples or whether the same sample was measured repeatedly
☐	☒	The statistical test(s) used AND whether they are one- or two-sided<br>*Only common tests should be described solely by name; describe more complex techniques in the Methods section.*
☐	☒	A description of all covariates tested
☐	☒	A description of any assumptions or corrections, such as tests of normality and adjustment for multiple comparisons
☐	☒	A full description of the statistical parameters including central tendency (e.g. means) or other basic estimates (e.g. regression coefficient) AND variation (e.g. standard deviation) or associated estimates of uncertainty (e.g. confidence intervals)
☐	☒	For null hypothesis testing, the test statistic (e.g. F, t, r) with confidence intervals, effect sizes, degrees of freedom and P value noted<br>*Give P values as exact values whenever suitable.*
☒	☐	For Bayesian analysis, information on the choice of priors and Markov chain Monte Carlo settings
☐	☐	For hierarchical and complex designs, identification of the appropriate level for tests and full reporting of outcomes
☒	☐	Estimates of effect sizes (e.g. Cohen's d, Pearson's r), indicating how they were calculated

Our web collection on statistics for biologists contains articles on many of the points above.

Software and code

Policy information about availability of computer code

Data collection	No software was used for data collection.
Data analysis	R(4.2); Matlab (R2018b); SPM12; CAT12

For manuscripts utilizing custom algorithms or software that are central to the research but not yet described in published literature, software must be made available to editors and reviewers. We strongly encourage code deposition in a community repository (e.g. GitHub). See the Nature Portfolio guidelines for submitting code & software for further information.

Data

Policy information about availability of data

All manuscripts must include a data availability statement. This statement should provide the following information, where applicable:

- Accession codes, unique identifiers, or web links for publicly available datasets
- A description of any restrictions on data availability
- For clinical datasets or third party data, please ensure that the statement adheres to our policy

The discovery cohort, Parkinson's Progression Marker Initiative (PPMI) database, is available on Parkinson’s Progression Markers Initiative www.ppmi-info.org/data. The external validation cohort, Fujian Medical University Union Hospital Parkinson's Disease (FJMUUH-PD) database, is available from the corresponding author upon request.

Research involving human participants, their data, or biological material

Policy information about studies with human participants or human data. See also policy information about sex, gender (identity/presentation), and sexual orientation and race, ethnicity and racism.

Reporting on sex and gender	N/A
Reporting on race, ethnicity, or other socially relevant groupings	N/A
Population characteristics	The discovery cohort was obtained from the Parkinson's Progression Marker Initiative (PPMI) database, whereas the external validation cohort was obtained from the Fujian Medical University Union Hospital Parkinson's Disease (FJMUUH-PD) database. The discovery cohort comprised 85 patients with PD, 25 FoG converters , and 60 FoG nonconverters. In the validation cohort, 55 patients, 18 FoG converters , and 37 FoG nonconverters were included for analysis.
Recruitment	Please see above.
Ethics oversight	The PPMI cohort was registered on ClinicalTrials.gov (NCT01141023), and the Ethical Standards Committee for Human Subjects approved the participating sites before the start of the study. Written informed consent was obtained from all study participants. The Ethics Committee of Fujian Medical University Union Hospital approved the FJMUUH-PD cohort (No. 2019-014), and participants provided written informed consent before enrollment, adhering to the Declaration of Helsinki.

Note that full information on the approval of the study protocol must also be provided in the manuscript.

Field-specific reporting

Please select the one below that is the best fit for your research. If you are not sure, read the appropriate sections before making your selection.

☒ Life sciences ☐ Behavioural & social sciences ☐ Ecological, evolutionary & environmental sciences

For a reference copy of the document with all sections, see nature.com/documents/nr-reporting-summary-flat.pdf

Life sciences study design

All studies must disclose on these points even when the disclosure is negative.

Sample size	The discovery cohort comprised 85 patients with PD, 25 FoG converters , and 60 FoG nonconverters. In the validation cohort, 55 patients, 18 FoG converters , and 37 FoG nonconverters were included for analysis.
Data exclusions	The exclusion criteria were patients under four years of follow-up, with incomplete follow-up, those who underwent DBS surgery during the follow-up, and those with diagnosed neurodegenerative diseases other than idiopathic PD during the follow-up.
Replication	Over 100 cross-validation iteration, we trained a random forest classifier in the training subset to estimate the contribution of each feature to the predictive accuracy of the model. Subsequently, we reconstructed and tested these probabilities in 100 resampled training and test datasets, evaluating the performance of each model using the area under the receiver operating characteristic curve (AUC).
Randomization	Randomization was not necessary as no intervention was carried out.
Blinding	Blinding was not necessary as no intervention was carried out.

Reporting for specific materials, systems and methods

We require information from authors about some types of materials, experimental systems and methods used in many studies. Here, indicate whether each material, system or method listed is relevant to your study. If you are not sure if a list item applies to your research, read the appropriate section before selecting a response.

Materials & experimental systems

n/a	Involved in the study
☒	☐ Antibodies
☒	☐ Eukaryotic cell lines
☒	☐ Palaeontology and archaeology
☒	☐ Animals and other organisms
☐	☒ Clinical data
☒	☐ Dual use research of concern
☒	☐ Plants

Methods

n/a	Involved in the study
☒	☐ ChIP-seq
☒	☐ Flow cytometry
☐	☒ MRI-based neuroimaging

Clinical data

Policy information about clinical studies
All manuscripts should comply with the ICMJE guidelines for publication of clinical research and a completed CONSORT checklist must be included with all submissions.

Clinical trial registration	Not applicable
Study protocol	Not applicable
Data collection	Not applicable
Outcomes	Not applicable

Plants

Seed stocks	Not applicable
Novel plant genotypes	Not applicable
Authentication	Not applicable

Magnetic resonance imaging

Experimental design

Design type	*Indicate task or resting state; event-related or block design.*
Design specifications	*Specify the number of blocks, trials or experimental units per session and/or subject, and specify the length of each trial or block (if trials are blocked) and interval between trials.*
Behavioral performance measures	*State number and/or type of variables recorded (e.g. correct button press, response time) and what statistics were used to establish that the subjects were performing the task as expected (e.g. mean, range, and/or standard deviation across subjects).*

Acquisition

Imaging type(s)	structural, diffusion
Field strength	1.5T; 3T
Sequence & imaging parameters	5.3.1 MRI metrics in PPMI<br>The 1.5-Tesla MRI scanners from various manufacturers, including SIEMENS, Philips Medical Systems, and GE Medical Systems, were used to acquire T1Wis<br>of 11 patients. The flip angle was 8–15º, and the matrix size was 192–516 pixels in the X and Y directions and 88–170 slices in the Z direction. The slice thickness was 1.0–1.5 mm, and the echo time (TE), inversion time (TI), and repetition time (TR) were 2.7–4.0 ms, 0 or 1,000.0 ms, and 7.0–8.5 or 2,400.0 ms, respectively. The 3.0-Tesla MRI scanners from the same manufacturers were used to acquire T1WIs of 74 patients. The flip angle was 9–13º, and the matrix size was 240.0–288.0 pixels in the X and Y directions and 152–192 slices in the Z direction. The slice thickness was 1.0–1.2 mm, and the TE, TI, and TR were 2.3–3.6 ms, 0, 450 or 900.0 ms, and 3.9–2,300.0 ms, respectively. The 3.0-Tesla MRI scanner

(SIEMENS TrioTim), with a flip angle of 90º, 64 gradient directions, and a matrix size of 1044.0 pixels in the X and Y directions and 65.0 slices in the Z direction, was used to acquire DTI images. The pixel size and slice thickness were 2.0 mm. The TE and TR were 88.0 and 670–9,300.0 ms, respectively.
5.3.2 MRI metrics in FJMUUH-PD
Brain imaging data were acquired using the GE 3.0-Tesla dual-gradient magnetic resonance scanner, and the sequences and scan parameters were as follows: a 3D cranial volume sequence (3D-BRAVO) obtained from high-resolution T1WI brain structure images, with TR = 8.7 ms, TE = 3.42 ms, TI = 400 ms, flip angle = 12º, matrix = 256 × 256, field of view = 240 × 240 mm, and 180 layers with a thickness of 1.1 mm. A spin echo-planar imaging sequence was used to obtain DTI scans for cross-sectional brain imaging, with the scanning level parallel to the anterior–posterior commissure. The DTI sequence required the following conditions: a TR of 6,000 ms, TE of 65.7 ms, flip angle of 90°, matrix of 128 × 128, a field of view of 240 × 240 mm, 55 layers with a thickness of 3 mm, continuous scanning without spacing, b-values of 0 and 1,000 s/mm2, and 16 nonlinear diffusion-sensitive gradient directions.

Area of acquisition: Whole brain scan

Diffusion MRI: ☒ Used ☐ Not used

Parameters: see above

Preprocessing

Preprocessing software: Matlab (R2018b); SPM12; CAT12

Normalization:
Cortical thickness analysis
The surface-based morphometry analysis was performed using Statistical Parametric Mapping software (SPM12; http://www.fil.ion.ucl.ac.uk/spm/software/spm12), extended by the Computational Anatomy Toolbox (CAT12; http://dbm.neuro.uni-jena.de/cat/). The CAT12 default settings were used as described in detail in the manual (http://dbm.neuro.uni-jena.de/cat12/CAT12-Manual.pdf) to estimate the cortical thickness. T1WI was registered with the Montreal Neurological Institute template, segmented into gray matter, white matter, and cerebrospinal fluid, and spatially normalized. A projection-based approach, which calculates the distance between the external cortical surfaces (the borders between the gray matter and cerebrospinal fluid), was used to estimate the left and right hemisphere cortical thicknesses. Finally, a 12-mm full width at the half maximum Gaussian kernel was used to smooth the cortical thickness.
DTI analysis
Sequential automated steps were performed using MATLAB (2018b) with Automatic Fiber Bundle Quantification (AFQ), an open-source software developed by Yeatman's team based on MATLAB that automatically identifies, quantifies, and analyzes white matter fiber pathways in the brain. The software automatically and efficiently extracts data on 20 major nerve fiber bundles across the brain and divides each into 100 isometric segments. Subsequently, FA and other diffusion tensor metrics are mapped to each segment to enable the accurate localization of abnormal changes in fiber bundle tensor imaging metrics35. AFQ is used to study white matter degeneration in mild cognitive impairment and its relationship with Alzheimer's disease36. Before running AFQ, the path should be confirmed, and anterior commissure (AC)–posterior commissure (PC) lines should be aligned with midsagittal planes by entering the relevant codes in the command window. This procedure aligns the AC (located below the anterior front of the vault), PC (located below the posterior thalamus and above the midbrain), and midsagittal planes in the image. After alignment, the software extracts the FA, axial diffusion, radial diffusion, MD, and other structural characteristic data. The specific procedure and white matter fiber analysis parameters are described in the methodology section of the supplemental file.

Normalization template: Specifications above (preprocessing software section)

Noise and artifact removal: Specifications above (preprocessing software section)

Volume censoring: Specifications above (preprocessing software section)

Statistical modeling & inference

Model type and settings:
We followed a feature selection approach using the random forest method to improve the predictive performance of the model. This method comprises several essential steps as follows. Initially, we randomly generated 100 training and divided the dataset into training and testing subsets, typically in a 3:1 ratio. Next, we used the 10-fold cross-validation process to ensure the robust evaluation of the model. The method was crucial because it could calculate feature importance. Over 100 cross-validation iteration, we trained a random forest classifier in the training subset to estimate the contribution of each feature to the predictive accuracy of the model. The computed feature importance accumulated across the cross-validation iterations. Features exceeding a predefined threshold, which was set at 50%, were deemed significant and subjected to further analysis. Next, the retained features were sorted based on their frequency of selection throughout the cross-validation process, ensuring that the most frequently selected and consistent features were prioritized.
We used the following four machine-learning methods: SVM with four different kernels (linear, polynomial, radial basis function (RBF), and sigmoid). When dealing with imbalanced datasets in the present study, we considered two distinct oversampling methods: ROSE and synthetic minority over-sampling technique (SMOTE). Finally, we compared the performance of these two models with and without feature selection to determine the most suitable approach for our data and task. This flexible approach allowed us to gain a better understanding of the data and optimize the modeling outcomes. A dataset was initially divided into a training set and a test set, with a ratio of 3:1, during machine-learning performance analysis. The training set was used for model construction and parameter tuning, whereas the test set was reserved for the final evaluation of the performance of the model. Further, to select the best model and fine-tune hyperparameters, we followed the 10-fold cross-validation method to assess the model on the training set. Ensuring the independence of the test

set from the training set and the cross-validation sets was crucial to guarantee the reliability of the final model evaluation. This workflow helped prevent overly optimistic estimations during evaluation because the test set remains separate from the model selection process. The machine-learning models were trained to predict the probability of FoG development for each patient in the test dataset, aiming to discover potential patient clusters.

Effect(s) tested: This is not relevant because this study is a structural MRI study rather than task fMRI

Specify type of analysis: ☐ Whole brain ☒ ROI-based ☐ Both

Anatomical location(s): Automatic Fiber Bundle Quantification;ROI_aparc_a2009s_thickness

Statistic type for inference (See Eklund et al. 2016): This is not relevant because MRI parameters was extract at ROI-evel

Correction: using the false-discovery rate (FDR) adjustment

Models & analysis

n/a	Involved in the study
☒	☐ Functional and/or effective connectivity
☒	☐ Graph analysis
☐	☒ Multivariate modeling or predictive analysis

Multivariate modeling and predictive analysis: Specifications above (Model type and settings)
